# Compositional Engineering of Ti_3_C_2_T_*x*_ MXene-NiMoO_4_ Hybrid Nanostructures for Enhanced Electrocatalytic Water Oxidation

**DOI:** 10.1021/acsaem.5c01467

**Published:** 2025-07-25

**Authors:** Saeed Sajjadi, Thorsten Schultz, Danielle A. Douglas-Henry, Karuppasamy Dharmaraj, Aline Alencar Emerenciano, Can Kaplan, Noel Marks, Kai S. Exner, Valeria Nicolosi, Norbert Koch, Michelle P. Browne

**Affiliations:** † Helmholtz Young Investigator Group Electrocatalysis: Synthesis to Devices, Helmholtz-Zentrum Berlin für Materialien und Energie GmbH, Albert-Einstein-Str. 15, 12489 Berlin, Germany; ‡ Centre for Functional and Surface Functionalized Glass, Alexander Dubček University of Trenčín, Trenčín 911 50, Slovakia; § 28340 Helmholtz-Zentrum Berlin für Materialien und Energie GmbH, Berlin 14109, Germany; ∥ Institut für Physik & CSMB, Humboldt-Universität zu Berlin, Berlin 12489, Germany; ⊥ School of Chemistry, CRANN and AMBER Research Centres, Trinity College Dublin, College Green, Dublin D02 PN40, Ireland; # University Duisburg-Essen, Faculty of Chemistry, Theoretical Catalysis and Electrochemistry, Universitätsstraße 5, 45141 Essen, Germany; ∇ Cluster of Excellence RESOLV, Bochum 44801, Germany; ○ Center for Nanointegration (CENIDE) Duisburg-Essen, Duisburg 47057, Germany

**Keywords:** NiMoO_4_ electrocatalyst, Ti_3_C_2_T_*x*_ MXene, oxygen evolution reaction (OER), water splitting, Operando Raman spectroscopy

## Abstract

A critical step in realizing the vision of green hydrogen through water splitting is to design oxygen evolution reaction (OER) catalysts that showcase a good balance of activity and stability. This work reports the compositional tuning of a NiMoO_4_ material and then the subsequent varying of Ti_3_C_2_T_*x*_ MXene with the NiMoO_4_ hybrid nanostructures as OER catalysts in alkaline media. In this work, the optimum NiMoO_4_ hybrid catalyst retained good stability over 24 h of chronopotentiometry on industrial relevant supports (Ni Felt) with an overpotential value of ca. 339 mV at 100 mA cm^–2^. Operando Raman spectroscopy revealed that catalytically active β-NiOOH species are formed during OER in NiMoO_4_ at lower overpotentials than for pure NiO and that a higher amount of the β-NiOOH was found in the 5% MXene loading. The ICP-OES analysis showed that Mo dissolution follows a volcano trend with MXene loading (peaking at 5 wt %) before decreasing at 10 wt %. Overall, these results hold great promises for rational design strategies for MXene-supported water oxidation catalysts in alkaline electrolytes.

## Graphical abstract

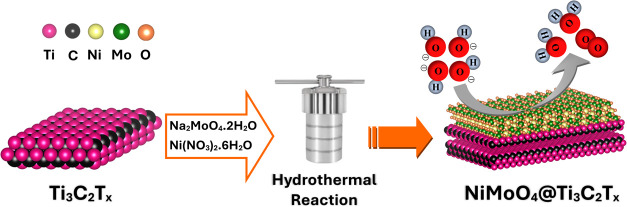

## 1 Introduction

The excessive use of fossil fuels generates substantial amounts of greenhouse gases, primarily CO_2_, leading to a continuous rise in atmospheric CO_2_ levels. This increase in CO_2_ levels impacts Earth’s climate and has devastating effects, as evidenced by the melting of Arctic ice and subsequent sea level rise.^1^ Researchers have recently prioritized carbon-neutral options over carbon-based ones in the quest for alternative fuels. Hydrogen (H_2_) has emerged as a promising alternative due to its high specific energy density (120–140 MJ/kg).

Various methods exist for hydrogen production, including metal hydride hydrolysis, steam reforming of fossil fuels, photoelectrochemical water splitting, and water electrolysis.^2^ Water electrolysis has gained significant attention for its environmentally friendly nature and zero carbon emissions. There are two half-cell reactions involved in water electrolysis: (I) the hydrogen evolution reaction (HER) at the cathode and (II) the oxygen evolution reaction (OER) at the anode. The conventional electrode potential for water splitting is 1.23 V vs RHE, determined based on thermodynamics; however, this value does not account for the reaction’s kinetic aspects, which differ according to the mechanistic stages of both OER and HER.^3^ These processes possess complex mechanisms with multiple electron transfers and various kinetic parameters. As a result, they require potentials that are always beyond the theoretical potential values and cannot proceed in the thermodynamically derived potentials. This additional potential beyond the theoretical value is known as ‘overpotential’.^3^ Minimizing this overpotential necessitates the development of suitable electrode materials for OER/HER that operate at low overpotentials under specific current densities. The efficiency of the OER significantly impacts the overall performance of electrolysis systems, which are crucial for clean energy solutions. Thus, optimizing and creating OER catalysts is vital for utilizing hydrogen as a clean energy carrier, facilitating large-scale, sustainable energy solutions.

Noble metal oxides (IrO_2_ and RuO_2_) are top-tier OER catalysts.^4^ Nevertheless, resource scarcity has challenged their industrialization, leading to rising expenses. As an alternative, transition metal oxides (TMOs) have recently gained attention for the OER due to their high stability, multiple oxidation states, abundance, and cost-effectiveness.^5^ In recent years, considerable studies have been conducted on transition metal oxides/hydroxides, including NiO,^6^ Co_3_O_4_,^7^ MnO_*x*_,^8^ and Ni­(OH)_2_,^9^ for OER. However, these oxides’ inherent semiconducting nature and high onset potential have limited their broader application.^10^ It has been observed that binary transition metal oxides have improved electrical conductivity, attributed to metal cation interactions that produce higher oxidation states, thus enhancing their electrochemical properties.^11^

Researchers have become increasingly interested in binary metal oxides with the general formula ABO_4_ (where A is Mn, Fe, Co, or Ni, and B is W or Mo) due to their unique physicochemical properties. Compared to monometallic metal oxides, these binary metal oxides have enhanced electrochemical activity as electrodes in energy storage devices and catalysis applications.^12^ One binary material that is not as widely studied as NiFe or NiCo oxides is NiMoO_4_. NiMoO_4_ offers many advantageous qualities as an electrocatalyst, such as low cost and the ability to tailor its three-dimensional morphology. Its hierarchical structures further increase both the surface area and the density of surface-active sites.

However, low electrical conductivity and structural discrepancies limit NiMoO_4_’s potential in energy conversion applications. Significant research efforts are needed to address these limitations and optimize the electrode material for long-term applications, as enhanced electron conductivity at the surface and interface boundaries improves electrochemical performance.^13^ One promising approach involves modifying NiMoO_4_ with conductive carbonaceous materials such as carbon nanotubes^14^ and graphene.^15^ Previous studies have shown that hybrid nanostructures can enhance electrochemical activity by improving reaction kinetics.^16,17^ However, using carbon-containing additives poses a challenge, as they may degrade when exposed to voltages typical for OER. This degradation can lead to additional anodic current, potentially skewing the analysis of OER performance.^18^

MXenes have garnered significant interest as potential support for improving metal oxide performance in OER catalysis.^19,20^ This is due to their unique blend of properties, including hydrophilicity, remarkable electrical conductivity (ranging from 8000 to 10,000 S/cm), and tunable surface chemistry.^21^ These two-dimensional materials, comprising transition metal carbides, nitrides, or carbonitrides, were first reported in 2011.^22^ The structure of MXenes features layers of M_6_X octahedra arranged in a hexagonal pattern. Their general formula is M_*n*+1_X*_n_* T_*x*_, where M stands for a transition metal element (such as Ti, V, or Nb), while X denotes either carbon or nitrogen. The value of n can range from 1 to 4, and the T_*x*_ component refers to the functional groups (such as −OH, –O, or –F) that terminate the outer layers of transition metals. The synthesis of MXenes typically involves the etching of the “A” (an element from group IIIA or IVA) layers from the MAX phases (M_*n*+1_AX*_n_*). Under the significantly stronger M–X bonds compared to the M–A bonds, it is possible to selectively chemically etch the A layers without causing damage to the M–X bonds. This process yields weakly bonded M_*n*+1_X*_n_* layers.^23^ The exfoliation conditions of these layers determine the nature of the surface terminations groups (T_*x*_).^24,25^ These surface groups can serve as attachment points for catalytically active substances. Specifically, −OH and –O terminations can establish robust chemical bonds with metal oxides. This bonding helps distribute metal nanoparticles evenly across the MXene surface, prevents them from agglomeration, and ensures a well-spread arrangement of catalytic sites. Moreover, these chemical interactions facilitate the transmission of electric charge between the MXene and the metal oxide particles, enhancing the final composite’s total conductivity and catalytic performance.^26^ Ti_3_C_2_T_*x*_, a titanium carbide variant, pioneered the way for many possible MXene compositions derived from the M_*n*+1_X*_n_* T_*x*_ formula.^22^ As the first successfully created MXene, it has naturally become a central focus of scientific inquiry. Various studies have highlighted its potential to support metal oxide-based OER catalysts. For example, combining Ti_3_C_2_T_*x*_ MXene with NiFeCe-layered double hydroxide (LDH) nanoflakes^27^ has produced catalysts that exhibit low overpotentials and improved charge transfer compared to the pure LDH. Meanwhile, Co_3_O_4_–RuO_2_/Ti_3_C_2_T_*x*_ ^28^ have shown better activity and stability in alkaline and acidic environments. Moreover, hypophosphite-intercalated FeNi (oxy)­hydroxide on V_2_C MXene^29^ has also displayed optimized intermediate adsorption and faster reaction kinetics. Even the straightforward mechanical mixing of Ti_3_C_2_T_*x*_ with Co­(OH)_2_ ^30^ has led to reduced overpotentials, highlighting the versatility and efficiency of MXene as a support for various metal oxide (hydroxide) OER catalysts. Notably, there appears to be a gap in the literature regarding the electrocatalytic properties of Ti_3_C_2_T_*x*_ MXene-supported NiMoO_4_ hybrid nanostructures for OER in an alkaline medium, presenting an opportunity for novel research in this area. The goal of this study is to show that combining NiMo oxides with MXenes enhances the OER activity compared to pure NiMo materials.

In this work, a range of novel Ti_3_C_2_T_*x*_ MXene-supported NiMoO_4_ hybrid composites was fabricated as OER electrocatalysts through a one-step hydrothermal method. To assess how the Ni/Mo ratio in NiMoO_4_ affects OER efficiency, various NiMoO_4_ samples with different Ni/Mo ratios were prepared. Furthermore, to examine the impact of Ti_3_C_2_T_*x*_ MXene content OER performance, the composites were prepared with differing quantities of Ti_3_C_2_T_*x*_.

## 2 Experimental Section

### 2.1 Materials

The raw materials were utilized without additional refinement. Sigma-Aldrich supplied sodium molybdate dihydrate (ACS grade, ≥99%), urea (ACS grade, ≥99%), 2-propanol (99.9%), potassium hydroxide (≥97%, pellet), and hydrochloric acid (37%). Thermo Fisher Scientific provided nickel­(II) nitrate hexahydrate (98%), lithium fluoride (300 mesh), and concentrated nitric acid (65%). Ti_3_AlC_2_ MAX phase precursor (∼40 μm) was purchased from Carbon (Ukraine). The nickel felt used in the experiments was obtained from Xinxiang AIDA Machinery Equipment Corporation in China. All experimental procedures employed ultrapure water with a resistivity of 18.2 MΩ·cm^–1^.

### 2.2 Preparation of Delaminated Ti_3_C_2_T_*x*_

Ti_3_C_2_T_*x*_ MXene was prepared through the in situ formation of HF to etch Ti_3_AlC_2_ MAX phase starting material. The synthesis began by combining 20 mL of 9 mol/L HCl with 2 g of LiF to create the etching solution. The mixture underwent stirring at ambient temperature for 30 min. Following this, 1 g of Ti_3_AlC_2_ was introduced slowly into the solution to avoid thermal runaway during the reaction. The etching process continued at 35 °C for 24 h. The resulting mixture underwent multiple washing cycles using ultrapure water and centrifugation at 3500 rpm for 5 min intervals to eliminate excess acid and reaction byproducts. Delamination began once the solution achieved neutrality (pH 7). The obtained slurry underwent dilution followed by 1 h of sonication treatment to ensure complete delamination. A final centrifugation step at 3500 rpm for 10 min separated the delaminated Ti_3_C_2_T_*x*_ MXene flakes in the supernatant.

### 2.3 Preparation of NiMo Oxides

Hydrothermal methods were employed to prepare nickel molybdate hydrate specimens. In a typical procedure, 2 mmol of nickel­(II) nitrate hexahydrate and 2 mmol sodium molybdate dihydrate were dissolved into 30 mL of ultrapure water. The solution was stirred for 15 min before being transferred into a Teflon-lined stainless-steel autoclave. This was then heated to 150 °C and maintained at that temperature for 6 h. The resulting powder was separated by centrifugation, rinsed five times with deionized water, and left to dry overnight in an oven set to 60 °C. Various nickel molybdate hydrate samples were produced using different Ni-to-Mo ratios. Specifically, the Ni/Mo ratios for samples NM1, NM2, and NM3 were 1:4, 1:1, and 4:1, respectively.

Samples of pure nickel oxide and molybdenum oxide were also prepared to enable a comparative assessment of the OER activity. For the nickel oxide synthesis, urea was used instead of sodium molybdate dihydrate. Similarly, concentrated nitric acid was used instead of nickel­(II) nitrate hexahydrate to prepare molybdenum oxide.

### 2.4 Preparation of NiMo Oxide/Ti_3_C_2_T_*x*_ Composites

To prepare NiMo oxide/Ti_3_C_2_T_*x*_ composites with varying concentrations of Ti_3_C_2_T_*x*_ (1, 2, 5, and 10 wt %), specific volumes of delaminated Ti_3_C_2_T_*x*_ solution (9 mg/L)—1.85, 3.67, and 11 mL—were first ultrasonically dispersed for 30 min. This dispersion was mixed with Ni/Mo (1:1) aqueous solution. After adding the Ni/Mo solution, the mixture underwent another 30 min dispersion process. All processes were conducted under a N_2_ atmosphere. The resulting suspension was transferred to a Teflon-lined stainless-steel autoclave and subjected to heat treatment at 150 °C for 6 h. The product was then collected via centrifugation, washed with deionized water five times, and dried in an oven at 60 °C overnight. From here on in, the prepared NiMo oxide/Ti_3_C_2_T_*x*_ composites will be referred to as 1% NM2T, 2% NM2T, 5% NM2T, and 10% NM2T, respectively, following the Ti_3_C_2_T_*x*_ weight percentages of 1, 2, 5, and 10%.

### 2.5 Characterization

X-ray diffraction (XRD) patterns were collected on a Bruker D8 ADVANCE X-ray diffractometer using Cu Kα radiation (λ = 1.5418 Å). Scans were performed at room temperature with a 0.02° step size across a 2θ range of 5 to 70°. A Zeiss MERLIN Scanning Electron Microscope (SEM) with a 0.1–30 keV field emission gun was used to examine the electrocatalysts’ morphologies at an accelerating 3–5 kV. Elemental mapping regions were imaged using Transmission Electron Microscopy (TEM, FEI Titan 80–300, Thermo Fisher Scientific) combined with an Energy Dispersive X-ray Spectroscopy (EDS, Bruker XFlash 6–30) detector. This was performed in Scanning Transmission Electron Microscopy (STEM) mode with a high-angle annular dark-field (HAADF) detector. X-ray photoelectron spectroscopy (XPS) analysis was carried out using a JEOL JPS-9030 system with a base pressure of 2 × 10^–9^ mbar. Samples were prepared by spreading the powders evenly on carbon tape. The system employed a nonmonochromatic Al source (300 W) for excitation and a hemispherical analyzer to detect photoelectrons, with pass energies of 50 eV for surveys and 20 eV for narrow scans, yielding a resolution of 1.1 eV. The binding energy scale of the analyzer was calibrated by setting the Au 4f_7/2_ and the Cu 2p_3/2_ peaks of clean gold and copper foils to 84.0 and 932.6 eV, respectively. However, since the samples showed charging, we shifted the binding energy of the C–C peak of the carbon tape to 285.0 eV or the C–Ti peak for the pristine Ti_3_C_2_ to 282.0 eV for comparison (see Figure S3 for C 1s spectra). CasaXPS was used to fit the Ti 2p spectra.^31^ Measurements for inductively coupled plasma optical emission spectroscopy (ICP-OES) were conducted with a Thermo Fisher iCAP 7400 DV in axial measurement mode.

ζ-Potentials were measured with a Malvern Panalytical Zetasizer Nano ZS. Dispersions containing 1 g/L of the material in a 1:1 mixture of DI water and isopropanol were sonicated for 1 h before injecting them into a disposable folded capillary cell (Malvern). For each sample, the average of 3 ζ-potential measurements was taken.

### 2.6 OER Activity

The OER activity of the samples was evaluated using a standard three-electrode setup in an alkaline solution at room temperature. A mercury/mercury oxide (Hg/HgO) electrode served as the reference, while a graphite rod was used as the counter electrode. The working electrode consisted of a catalyst-loaded glassy carbon (GC) electrode with a 2 mm diameter and 0.0314 cm^2^ surface area. A PalmSens (Netherlands) electrochemical workstation was used for measurements. Catalyst inks were prepared by mixing 10 mg of catalyst powder with 1 mL of a 1:1 (vol.) DI water/isopropanol solution and 8 μL of Nafion, followed by 15 min ultrasonication. To achieve a 0.32 mg·cm^–2^ catalyst loading, 1 μL of ink was applied to the polished GC electrode and air-dried at room temperature. All tests were conducted in nitrogen-saturated 1 mol·L^–1^ NaOH electrolyte. The potentials measured against the Hg/HgO reference electrode were converted to the reversible hydrogen electrode (RHE) scale using the Nernst equation

1 $$E_{\mathrm{RHE}}=E_{\mathrm{Hg}/\mathrm{HgO}}+0.059\mathrm{pH}+E_{\mathrm{Hg}/\mathrm{HgO}}^{0}$$

In this equation, *E*_RHE_ represents the potential versus RHE, *E*_Hg/HgO_ is the experimentally measured potential against the Hg/HgO reference electrode, pH is the electrolyte’s pH value, and *E*°_Hg/HgO_ (0.098 V) is the standard potential of the Hg/HgO reference electrode at 25 °C.

Before other measurements, cyclic voltammetry (CV) was performed for 8 cycles between −0.2 and 0.4 V at 40 mV·s^–1^ to activate the catalyst. Linear sweep voltammetry (LSV) and Tafel slope measurements were performed from 0 to 0.8 V (vs Hg/HgO) at 1 mV·s^–1^. IR-drop corrections were applied using the *R*_u_ values obtained from electrochemical impedance spectroscopy (EIS) at −0.2 V vs Hg/HgO. A 90% compensation (*E*_corrected_ = *E*_measured_ – 0.9 × *I* × *R*_u_) was applied to all LSV and Tafel data sets. The *R*_u_ values for each sample are listed in Table S2. For operando Raman measurements, no IR correction was applied to the electrochemical potentials to preserve accurate voltage control during spectral acquisition. Charge transfer resistance (*R*_ct_) was measured via EIS at 0.7 V vs Hg/HgO. Double-layer capacitance (*C*_dl_) was determined by conducting multiple CVs over a 100-mV range at scan rates from 10 to 200 mV·s^–1^. Currents from the non-Faradaic region were plotted against the scan rate, with the slope indicating the capacitance. To estimate the electrochemically active surface area (ECSA), a specific capacitance value of 40 μF·cm^–2^ was utilized. This approach involves dividing the measured *C*_dl_ by the specific capacitance to calculate the ECSA of the catalyst materials. Additionally, to determine the cell’s resistance, EIS was recorded over a frequency range of 1 Hz to 1 MHz, with an oscillation amplitude of 10 mV in a non-Faradaic region.

The catalyst was spray-coated onto a 1 cm^2^ nickel felt (NF) electrode to assess scalability and long-term stability, and chronopotentiometry was performed at 10 and 100 mA·cm^–2^ for 24 h. Before applying the catalyst, the NF underwent a cleaning process. It was first sonicated in 35% hydrochloric acid for 5 min to eliminate surface oxide films. Subsequently, the NF was subjected to sequential sonication treatments in acetone, ethanol, and deionized water, each lasting 5 min. The cleaned NF was dried in an oven for 12 h. 1.0 mg cm^2^ powder NiO catalysts were loaded on Ni felt. For the NiFeOOH on Ni felt, electrodeposition was performed at −10 mA cm^–2^ until it reaches the loading of 1.0 mg cm^2^. Ni foil is used as counter electrode. The electrolyte bath contained 0.1 M NiNO_3_, 0.1 M FeNO_3_, and 0.5 M Boric acid. After the electrodeposition, samples were dried at 80 °C for 4 h. Before the LSV measurement, deposited sample was electrochemically activated by cyclic voltammetric technique for 10 cycles between 0 to 1.0 V Hg/HgO at 200 mV s^–1^. The stability tests of NF samples were conducted at 250 rpm stirring rate using three electrode configuration consisting of catalyst coated electrode, graphite rod, and Hg/HgO (1.0 M NaOH) as working, counter, and reference electrodes, respectively.

### 2.7 Operando Raman

Operando Raman spectroscopic investigations were performed utilizing an i-Raman Plus 532H portable spectrometer equipped with a 532 nm laser integrated with a PalmSens electrochemical workstation. The potential-dependent operando Raman analysis employed a magnetic-mount Raman electrochemical flow cell (Raman ECFC 3.5 cm^2^, Redox.me) containing 4.5 mL of NaOH (1 M) electrolyte solution. A catalyst sample was deposited via spray coating onto the surface of a gold support, which functioned as an electrical contact for the working electrode. The experimental setup incorporated a platinum wire as the counter electrode and a mercury/mercury oxide (Hg/HgO) electrode as the reference electrode. Chronoamperometric measurements were executed across potential ranges from 1.30 V (vs RHE) to 1.6 V (vs RHE) during the operando Raman characterization. Each potential was sustained for 30 s, followed by acquiring a sequence of 5 Raman spectra over 5 min. While Raman spectra were collected across the entire spectral range of 0–3400 cm^–1^, the analysis focused exclusively on the fingerprint region (100–1000 cm^–1^), as this domain revealed critical spectral peaks indicating material structural transformations.

### 2.8 DFT Study of OER Mechanisms on Ti_3_C_2_T_*x*_ MXene

To gain insight into the OER on Ti_3_C_2_T_*x*_ at the atomic level, electronic structure calculations were performed within the framework of density functional theory (DFT). The computational hydrogen electrode approach^32^ was employed to determine the free-energy changes of the elementary steps of the OER, which were evaluated through descriptor-based analysis using the *G*_max_(U) descriptor.^33,34^ Computational details are provided in the Supporting Information.

## 3 Results and Discussion

A series of NiMoO_4_ and NiMoO_4_/Ti_3_C_2_T_*x*_ hybrid materials were synthesized using a facile hydrothermal method, as illustrated in Figure 1. Initially, Ti_3_C_2_T_*x*_ MXene nanosheets were prepared through selective etching of the Al layer from Ti_3_AlC_2_ MAX phase using in situ HF formation, followed by sonication-assisted delamination to obtain dispersed Ti_3_C_2_T_*x*_ nanosheets. The synthesis of NiMoO_4_ with different Ni/Mo ratios (1:4, 1:1, and 4:1) was achieved using nickel nitrate and sodium molybdate precursors under hydrothermal conditions at 150 °C for 6 h. Consequently, NiMoO_4_/Ti_3_C_2_T_*x*_ composites were prepared by incorporating varying amounts of Ti_3_C_2_T_*x*_ nanosheets (1–10 wt %) into the optimized Ni/Mo (1:1) precursor solution and hydrothermal treatment. For simplicity, the binary metal oxides are titled NM1, NM2, and NM3 (based on Ni/Mo ratios), while the MXene-containing composites are referred to as 1% NM2T, 2% NM2T, 5% NM2T, and 10% NM2T according to their Ti_3_C_2_T_*x*_ content.

**Figure 1:**
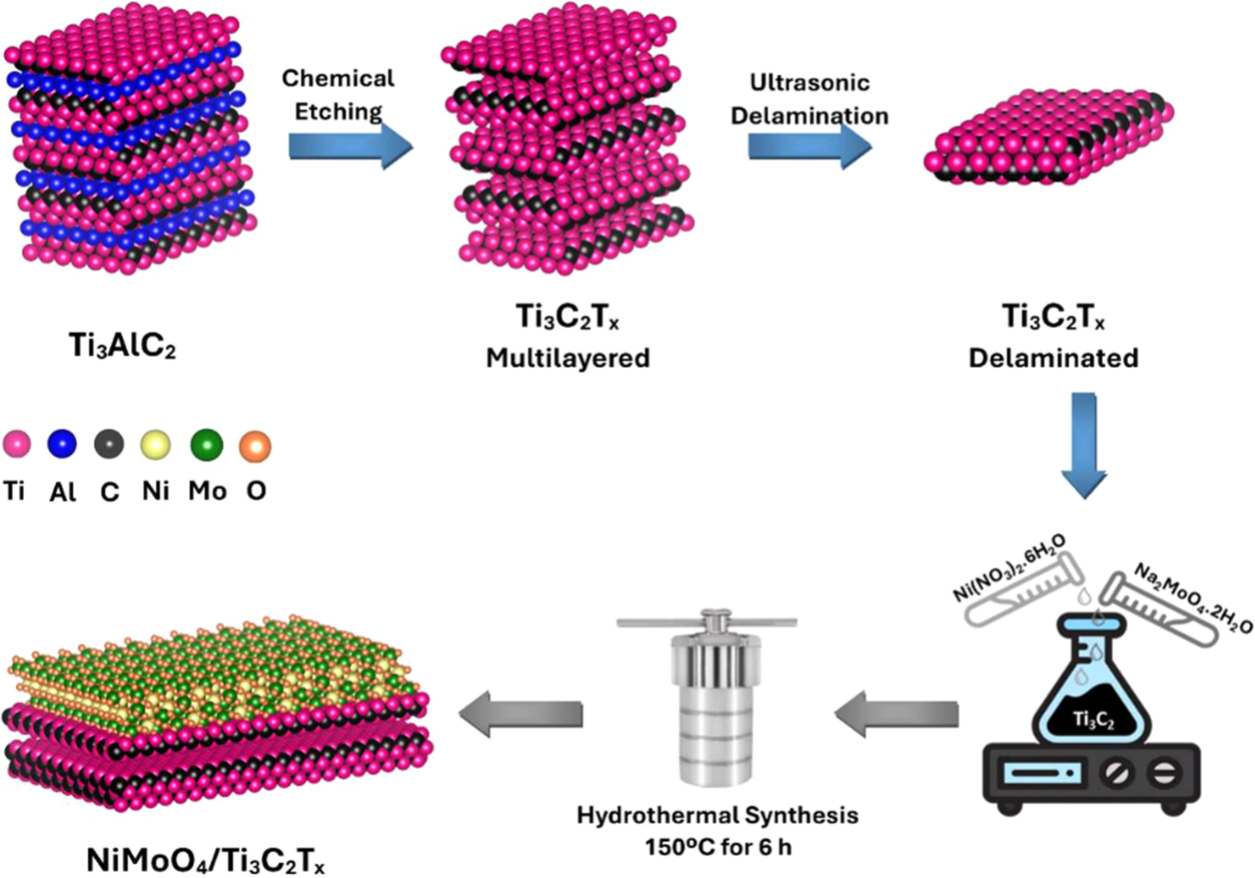
Schematic representation of the synthesis process for NiMoO_4_/Ti_3_C_2_T_*x*_ composites.

The morphological characteristics of the prepared samples were investigated using SEM (Figure 2). The pure NiO sample (Figure 2a) exhibited distinctive microspherical particles with diameters ranging from 1 to 3 μm. Notably, these microspheres were decorated with smaller nanospheric particles on their surface, creating a hierarchical structure. In contrast, pristine MoO_3_ displayed a rod-like morphology, with nanorods measuring approximately 100–190 nm in diameter and extending several micrometers (1–3 μm) in length (Figure 2b). The influence of varying Ni/Mo ratios on morphological evolution was particularly interesting. In the Mo-rich sample (NM1, Ni/Mo 1:4), the nanorod morphology persisted, reminiscent of pure MoO_3_, albeit with slightly reduced diameters (Figure 2c). As the nickel content increased in NM2 (Ni/Mo 1:1) while maintaining the rod-like structure, a significant reduction in both the diameter and length of the nanorods was observed compared to pristine MoO_3_ (Figure 2d). A dramatic morphological transformation occurred in the Ni-rich sample (NM3, Ni/Mo 4:1), where the structure reverted to spherical particles similar to NiO, but with diameters ranging from 0.5 to 1.5 μm (Figure 2e). Unlike the parent NiO structure, these spheres featured distinctive nanoflakes on their surface. The systematic combination of Ni and Mo generally reduced particle dimensions compared to their single-metal oxide counterparts. Among all synthesized samples, NM2, with its equimolar Ni/Mo ratio, demonstrated the smallest particle size, suggesting enhanced surface area availability. This observation indicates that the synergistic interaction between Ni and Mo at this specific ratio promotes the formation of finer structures.

**Figure 2:**
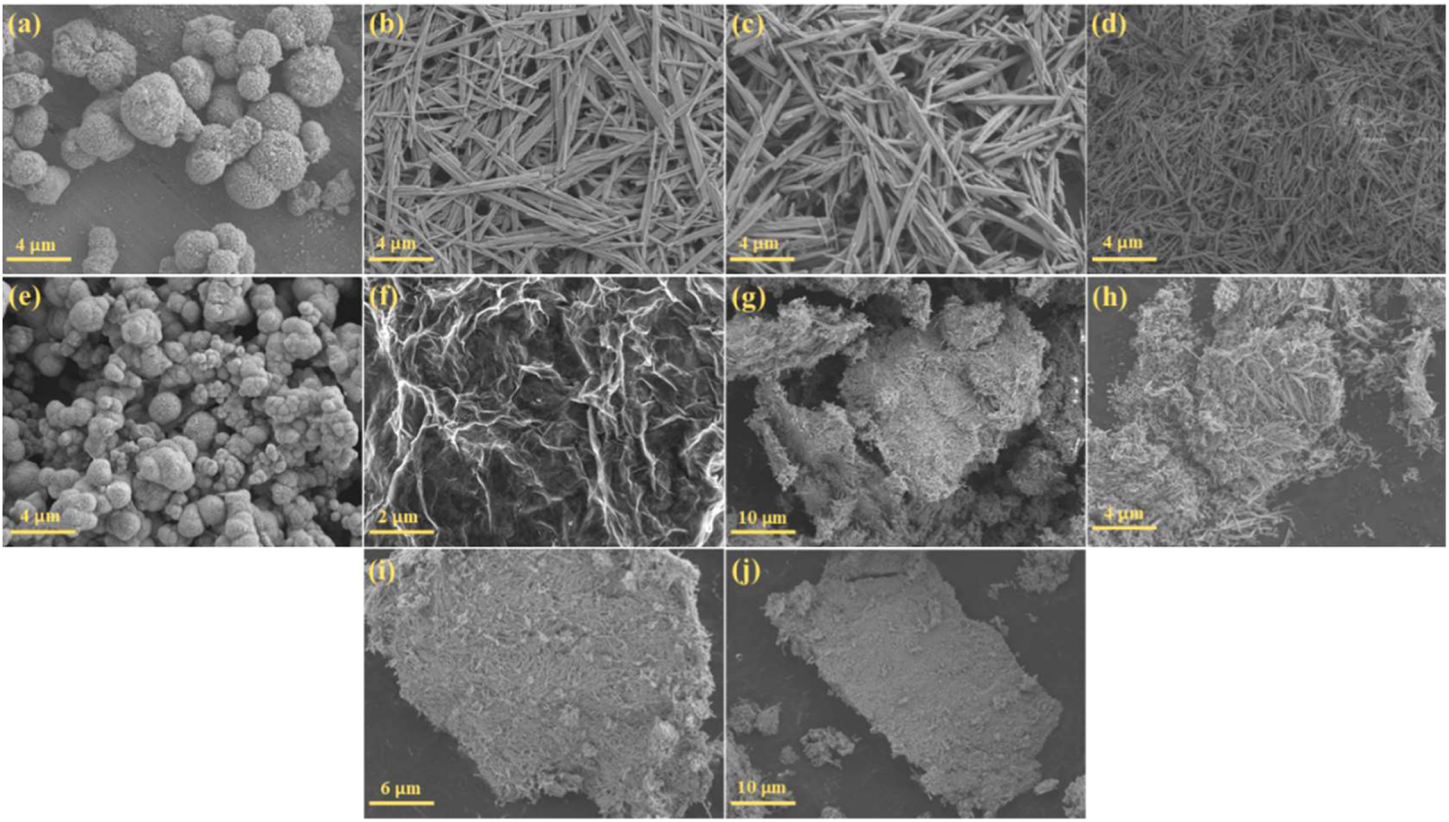
SEM images of (a) pure NiO, (b) pure MoO_3_, (c) NM1, (d) NM2, (e) NM3, (f) Ti_3_C_2_T_*x*_, (g) 1% NM2T, (h) 2% NM2T, (i) 5% NM2T, and (j) 10% NM2T.

Furthermore, the morphological characteristics of Ti_3_C_2_T_*x*_ MXene nanosheets and their composites with NM2 were examined. The SEM images of pristine Ti_3_C_2_T_*x*_ MXene revealed well-defined 2D nanosheet structures with a layered architecture, where sheets were stacked upon each other (Figure 2f). The absence of bulk particles confirmed the successful etching and delamination of Ti_3_AlC_2_ precursor into Ti_3_C_2_T_*x*_ MXene nanosheets. Incorporating NM2 with Ti_3_C_2_T_*x*_ MXene at varying concentrations (1, 2, 5, and 10%) resulted in distinct morphological features. In the composites containing 1 and 2% Ti_3_C_2_T_*x*_ (1% NM2T and 2% NM2T), the NM2 nanorods were abundantly distributed across the MXene nanosheet surfaces, creating a dense coverage pattern (Figure 2g,h). Interestingly, at 5% Ti_3_C_2_T_*x*_ loading (5% NM2T), more uniform and smoother coverage of NM2 nanorods was observed on the MXene surface (Figure 2i). The 10% NM2T showed reduced NM2 nanorod coverage compared to 5% NM2T (Figure 2j). A notable observation across all composite samples was the role of Ti_3_C_2_T_*x*_ MXene nanosheets as an effective support material. The presence of these nanosheets significantly mitigated the agglomeration tendency of NM2 nanorods, leading to better particle dispersion. This improved distribution of NM2 nanorods on the MXene surface suggests enhanced surface area availability, which could benefit various applications.

Transmission Electron Microscopy (TEM) with Energy Dispersive X-ray Spectroscopy (EDS) mapping was conducted to confirm the elemental composition of both the pure and composite materials. The EDS results show that all samples contain Ni and Mo, with Ti also present in all composite materials, Figure S4. EDS mapping of NM2 (Figure S5) indicates a good distribution of Ni and Mo within the rod-like structure of NM2. Additionally, the EDS mapping of the composites highlights a good distribution of Ni and Mo. It reveals that a notable portion of the surface of the MXene nanosheets is covered by NM2 rods, as shown in Figure S5, for the 1% NM2T, 2% NM2T, 5% NM2T, and 10% NM2T respectively.

X-ray diffraction analysis was used to investigate the crystalline structures of the synthesized materials (Figure 3). The XRD pattern of molybdenum oxide (Figure 3a) showed sharp diffraction peaks at 2θ = 12.8 and 25.74°, which are characteristic of the orthorhombic α-MoO_3_ phase. Additional reflections were observed at 23.38, 27.2, 39, 46.12, and 58.8°, further confirming the formation of crystalline MoO_3_.^35^ The nickel oxide sample exhibited diffraction peaks at 37, 43.2, and 62.5°, corresponding to the (111), (200), and (220) planes, indicating the formation of NiO.^36^ Distinct peaks at 14.9, 26, and 40.1° suggested the presence of nickel hydroxy-carbonate (Ni­(HCO_3_)_2_) species, likely due to partial carbonation during the hydrothermal synthesis.^37^ Building upon these single-oxide systems, the mixed Ni–Mo oxide samples showed distinct variations with changing Ni/Mo ratios. In the Mo-rich sample (NM1), the peaks at 9.4, 28.4, and 34.09° indicated the formation of the NiMoO_4_ phase. The equimolar sample (NM2) exhibited increased crystallinity with characteristic NiMoO_4_ peaks at 10.05, 13.56, 27.21, and 29.69°. The Ni-rich sample (NM3) showed sharp, high-intensity peaks at 9.74 and 13.42°, indicating the formation of a well-crystallized phase. The persistence of strong diffraction peaks at 27.21 and 29.69°, albeit with different relative intensities from NM2, suggests maintaining the NiMoO_4_ structure even at higher Ni content, likely accompanied by NiO-rich domains.^38,39^

**Figure 3:**
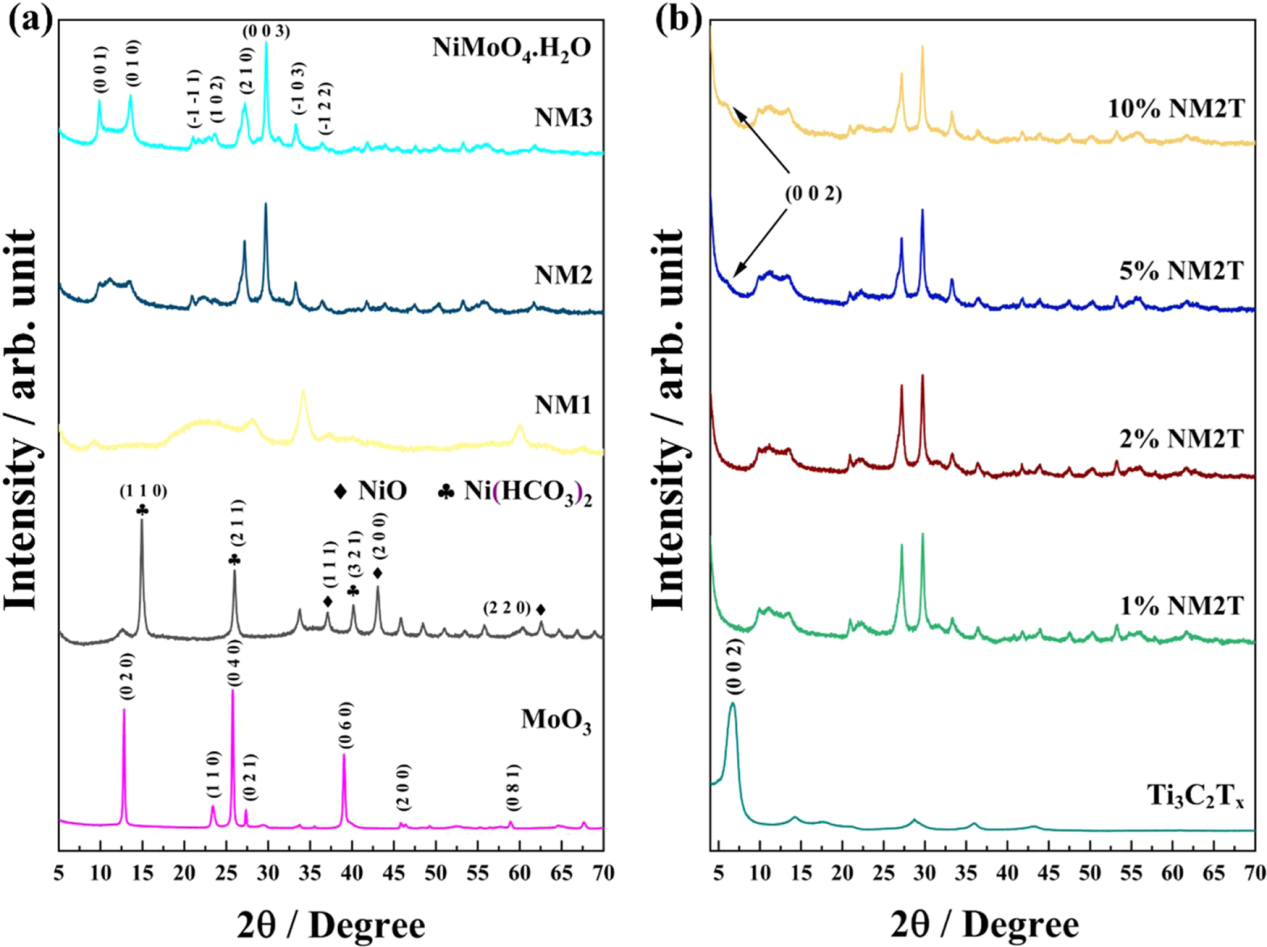
XRD patterns of (a) single and multimetal oxides with varying Ni/Mo ratios; and (b) Ti_3_C_2_T_*x*_ along with NiMo/Ti_3_C_2_T_*x*_ compositions featuring different Ti_3_C_2_T_*x*_ content.

Parallel to the oxide system development, the XRD patterns provided clear evidence of successful synthesis and transformation from Ti_3_AlC_2_ MAX phase to Ti_3_C_2_T_*x*_ MXene nanosheets, with subsequent structural modifications during hydrothermal treatment. The parent Ti_3_AlC_2_ MAX phase (Figure S6) showed characteristic peaks at 9.51 and 38.72°, corresponding to (002) and (104) planes, respectively.^40^ Upon etching, the (002) peak shifted to 6.69°, indicating successful exfoliation and increased interlayer spacing. The peak broadening observed across all reflections, particularly in the peaks at 14.23, 28.72, and 35.9°, suggests the formation of few-layer MXene sheets. The disappearance of the sharp MAX phase peaks confirms the effective removal of the aluminum layer during the etching process.^41,42^ As a reference for the metal oxide/MXene composite materials, hydrothermal treatment of the bare Ti_3_C_2_T_*x*_ MXene nanosheets was carried out and resulted in partial structural changes (Figure S7). This is evidenced by the emergence of anatase TiO_2_ (25.26°), suggesting partial oxidation of MXene sheets under hydrothermal conditions and expanded interlayer spacing due to water intercalation (peaks at 4.73 and 6.12°).^43,44^ In the 1% NM2T sample (Figure 3b), the diffraction pattern largely resembled that of the parent NM2 material, showing characteristic NiMoO_4_ peaks at 27.12 and 29.67°, along with the triplet peaks at 9.9, 11.15, and 13.41°. Increasing MXene content to 5 and 10% led to the emergence of the characteristic MXene (002) peak at 6.02° while preserving the oxide structure. The progressive intensification of this peak from 5 to 10% loading indicates the successful incorporation of MXene sheets while maintaining their layered structure. Despite the expected partial oxidation of Ti_3_C_2_T_*x*_ MXene to TiO_2_ under hydrothermal conditions, the characteristic anatase peak was not detectable in the composite patterns, likely due to the dominant diffraction intensity of the NiMoO_4_ phase and the relatively low concentration of any TiO_2_ formed during the synthesis.

The Raman spectra of the NiMo oxide samples exhibit characteristic vibrational modes associated with the NiMoO_4_ structure (Figure 4a). NM3 displays a prominent peak at 945.8 cm^–1^, attributed to the symmetric stretching mode of Mo=O bonds. The corresponding asymmetric stretching vibrations are observed at 862.7 and 827.4 cm^–1^, while the band at 353.3 cm^–1^ corresponds to the Mo–O bending mode. Similar spectral features are observed in the NM2, indicating the preservation of the primary NiMoO_4_ structure. Interestingly, in the NM1, a slight shift in peak positions is observed. The symmetric Mo=O stretching mode shifts to a lower wavenumber at 936.3 cm^–1^, accompanied by corresponding shifts in the asymmetric stretching modes to 853.2 and 798.6 cm^–1^. The Mo–O bending mode also exhibits a shift to 343.4 cm^–1^. These systematic peak shifts with increasing Mo content suggest subtle modifications in the local coordination environment of the Mo centers, likely due to the increased influence of neighboring Mo atoms. All observed vibrational modes agree with previously reported Raman spectra for NiMoO4 compounds in the literature.^15,45,46^

**Figure 4:**
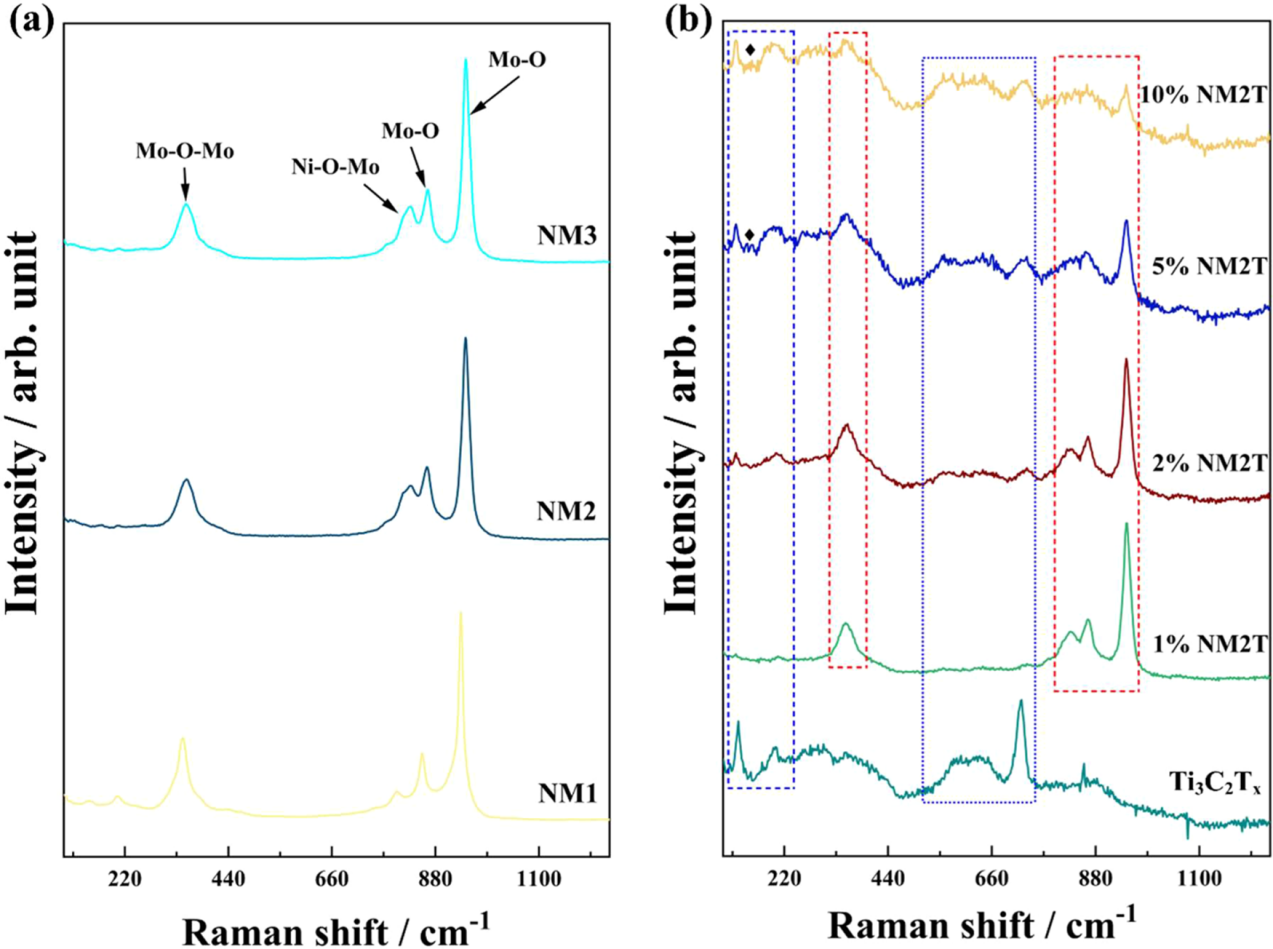
Raman spectra of (a) multimetal oxides with varying Ni/Mo ratios; and (b) Ti_3_C_2_T_*x*_ along with NiMoO_4_/Ti_3_C_2_T_*x*_ compositions featuring different Ti_3_C_2_T_*x*_ content. The blue boxes highlight the Raman peaks related to the Ti_3_C_2_T_*x*_ material; the red boxes indicate the peaks associated with the NiMoO_4_ component.

The pristine Ti_3_C_2_T_*x*_ material (Figure 4b) exhibits sharp features at 122.4, 203.4, 585–650 (a broad peak), and 720.5 cm^–1^, which correspond to the in-plane (E_1g_) and out-of-plane (A_1g_) vibrational modes of Ti and C atoms, as well as the stretching vibrations of surface functional groups such as Ti–O and Ti–OH, along with the Ti–C stretching mode (A_1g_).^47–49^ In the 1% NM2T sample, the Raman spectrum is dominated by the characteristic peaks of the underlying NM2 structure. The NM2 vibrations, such as the symmetric Mo=O stretching at 945.8 cm^–1^ and the asymmetric Mo=O modes at 862.7 and 827.4 cm^–1^, maintain high intensity. However, the Ti_3_C_2_T_*x*_-related peaks are barely discernible, indicating that the Ti_3_C_2_T_*x*_ content is too low to influence the overall Raman signature significantly. As the Ti_3_C_2_T_*x*_ content increases to 2% in the NM2T sample, the NM2 peaks remain the most prominent features, but the Ti_3_C_2_T_*x*_ contributions start to become more visible. The characteristic Ti_3_C_2_T_*x*_ bands, though still relatively weak, can be observed with slightly higher intensity than the 1% NM2T sample. The 5% NM2T and 10% NM2T samples show a more pronounced effect of the increasing Ti_3_C_2_T_*x*_ additive. While the NM2 peaks are still present, their relative intensities diminish as the Ti_3_C_2_T_*x*_ features become more dominant. Interestingly, in these higher Ti_3_C_2_T_*x*_ content samples, a small peak emerges at 149.5 cm^–1^, which may be attributed to the formation of Ti–O species due to the partial oxidation of Ti_3_C_2_T_*x*_ during the hydrothermal synthesis, which was also observed for the bare Ti_3_C_2_T_*x*_ from XRD, Figure S7.^50^

XPS was carried out to give insight into the surface chemical states. The Mo 3d spectrum of the pristine and composite containing Mo (Figure 5a) showed characteristic Mo 3d_5/2_ and Mo 3d_3/2_ peaks at ca. 233 eV and ca. 236 eV, respectively, confirming the presence of molybdenum in its Mo^6+^ oxidation state.^51^ In the Ni 2p region (Figure 5b), the pure and composite Ni containing materials showed a characteristic Ni 2p_3/2_ peak at ca. 857 eV with a satellite feature associated with Ni­(OH)_2_ and NiMoO_4_, respectively.^52,53^

**Figure 5:**
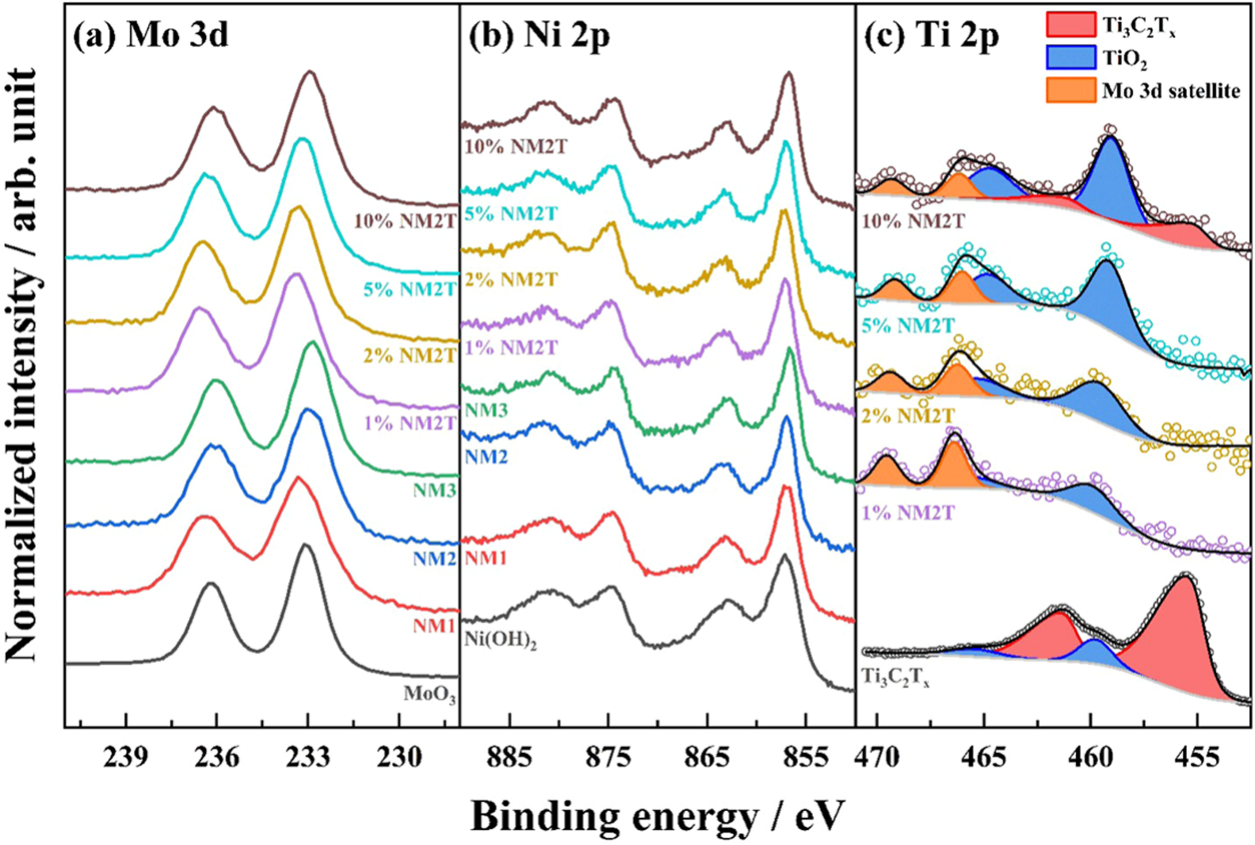
XPS core level spectra of (a) Mo 3d, (b) Ni 2p, and (c) Ti 2p regions for the synthesized catalysts.

The Ti 2p spectra (Figure 5c) provided additional insights into MXene integration and surface chemistry. The pure Ti_3_C_2_T_*x*_ showed characteristic MXene Ti–C peaks at 455.6 and 461.4 eV, along with TiO_2_-related signals at 459.8 and 465.5 eV, indicating slight surface oxidation.^54^ However, at the surface of the composite materials, TiO_2_ is mainly present, in agreement with the oxidation of the MXenes during the hydrothermal synthesis as observed by XRD and Raman.

The effect of Ti_3_C_2_T_*x*_ MXene incorporation on the surface charge of a NiMo catalyst was investigated by ζ-potential measurements (Figure S8). Pure Ti_3_C_2_T_*x*_ exhibited a negative ζ-potential of – 8.31 mV, attributable to surface terminations such as −O, −OH, and −F.^55^ The pure NiMo catalyst showed a less negative ζ-potential of −3.07 mV, which can be attributed to negatively charged surface oxides or hydroxides of nickel and molybdenum.

Upon incorporation of 1 wt % Ti_3_C_2_T_x,_ (1% NM2T) the ζ-potential shifted slightly to −3.29 mV, suggesting minimal impact on the surface charge and a predominant NiMo surface character. At 2 wt % (2% NM2T), the ζ-potential became more negative (−4.75 mV), indicating a more significant contribution from the MXene surface. Interestingly, 5% NM2T showed a slightly less negative value of −4.00 mV, possibly indicating an increased surface interaction between the MXene and the NiMo catalyst at this MXene content which could lead to partial charge neutralization. The closer to neutral surface charge in this composition may facilitate more efficient ion transport at the electrode/electrolyte interface, by reducing electrostatic repulsion for anionic species (e.g., OH^–^) while still maintaining colloidal stability. These results are in good agreement with the SEM analysis (Figure 2g–j) which revealed that composite 5% NM2T exhibited a more uniform and smoother coverage of NiMo nanorods on the MXene surface, in contrast to the dense, possibly less-accessible coverage seen at lower loadings. This balanced interfacial environment, marked by optimal surface potential and improved morphological homogeneity, likely contributes to enhanced reactant accessibility and charge transfer. At 10 wt % Ti_3_C_2_T_x,_ the ζ-potential further decreased to −7.30 mV, resembling that of pristine MXene, while SEM revealed reduced nanorod coverage, suggesting a decline in the beneficial interfacial configuration found at 5 wt %.

### 3.1 Electrocatalytic OER Activity

The OER activity of the synthesized catalysts was initially evaluated using a three-electrode configuration in N_2_-saturated 1.0 M NaOH electrolyte with a flat glassy carbon working electrode. LSV measurements revealed distinct differences in catalytic activity among the monometallic and binary oxide catalysts (Figure 6a). Among the monometallic oxides, NiO demonstrated moderately higher catalytic activity than MoO_3_. The low current response of the MoO_3_ can be attributed to the inherently poor OER activity of MoO_3_ in alkaline media. The incorporation of Mo into the NiO structure significantly influenced the catalytic performance, with the effect highly dependent on the Ni/Mo ratio. The optimal composition was achieved in NM2, which exhibited an increased OER activity compared to the two parent and two other NiMoO_4_ catalysts, requiring an overpotential of 491 mV to achieve the benchmark current density of 10 mA·cm^–2^. This significant enhancement in catalytic activity, compared to other compositions, suggests a synergistic effect between Ni and Mo at this specific ratio. The lower performance of NM1 compared to NM2 suggests that excess Mo content may not be beneficial for OER activity, possibly due to the dominance of the less active MoO_3_ phase. Conversely, NM3 showed improved activity over NM1. Still, it did not match the performance of NM2, indicating that while a higher Ni content is generally favorable for OER, the optimal synergistic effect is achieved at the equimolar ratio. Furthermore, from the Raman Spectroscopy, Figure 4, the NM1 materials had a slightly modified NiMoO_4_ structure compared to the NM2 and NM3, more than likely due to the increased amount of Mo in the NM1.

**Figure 6:**
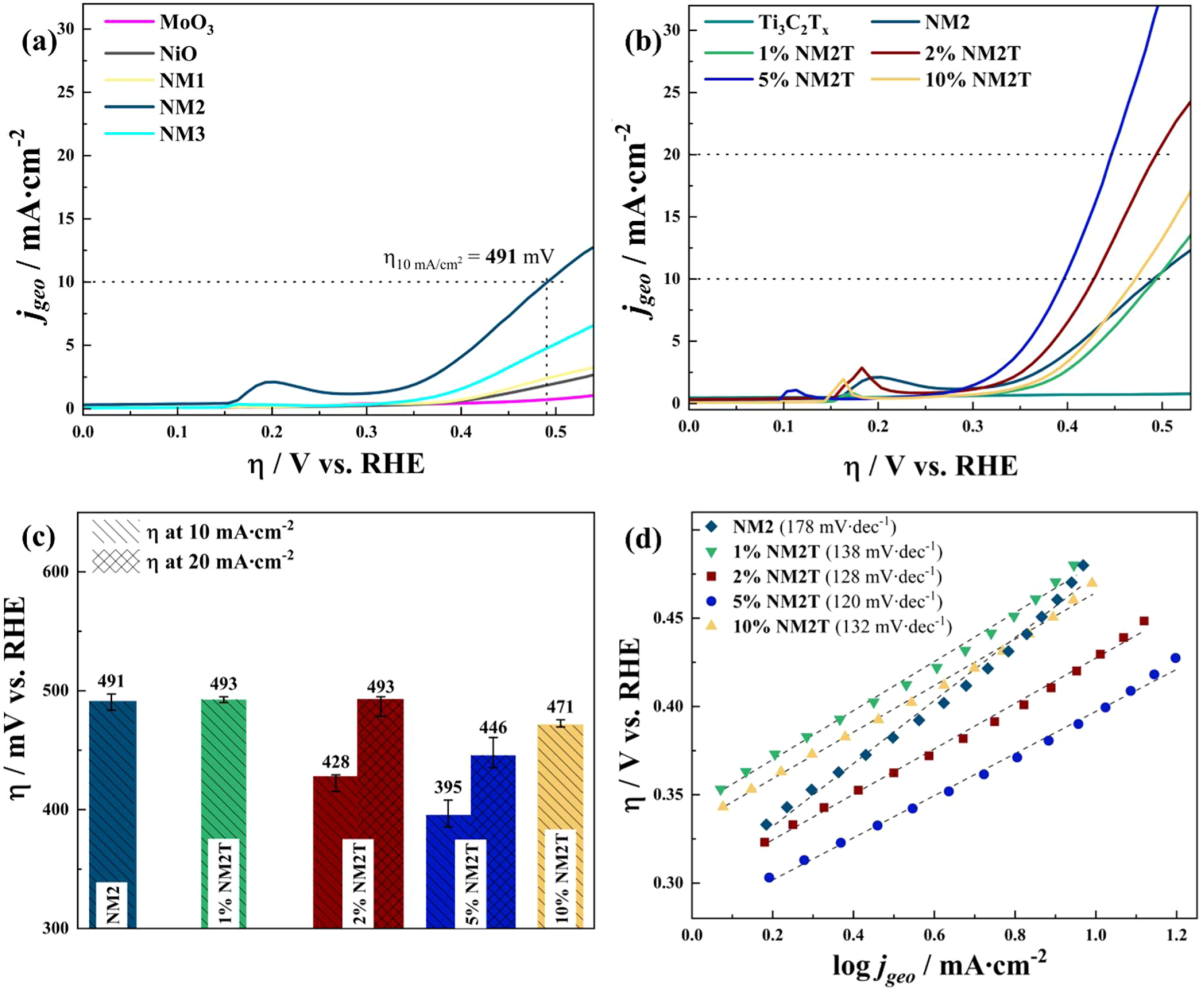
*iR*-corrected geometric area-normalized LSV curves for (a) the single and multimetal oxide samples; and (b) the Ti_3_C_2_T_*x*_ and NiMoO_4_/Ti_3_C_2_T_*x*_ compositions featuring different Ti_3_C_2_T_*x*_ contents. (c) Geometric area-normalized overpotential values at 10 and 20 mA·cm^–2^. (d) Tafel plots of NM2 and NiMoO_4_/Ti_3_C_2_T_*x*_ compositions.

Building upon the superior performance of NM2, the effect of incorporating Ti_3_C_2_T_*x*_ MXene as a conductive support material at various weight loadings was investigated (Figure 6b). While pure Ti_3_C_2_T_*x*_ MXene exhibited negligible OER activity, its integration with NM2 led to remarkable improvements in catalytic performance, with the effect being highly dependent on the MXene loading. The electrochemical measurements revealed a nonlinear relationship between MXene content and OER activity. At 1 wt % loading (1% NM2T), the composite showed comparable performance to pristine NM2, requiring an overpotential of 493 mV to achieve 10 mA·cm^–2^. A significant enhancement was observed with 2% NM2T, which only needed 428 mV overpotential for the same current density. The optimal performance was achieved with 5% NM2T, demonstrating an exceptionally low overpotential of 395 mV at 10 mA·cm^–2^, representing a substantial improvement of 96 mV compared to the pristine NM2 catalyst. Notably, only 2% NM2T and 5% NM2T could sustain current densities up to 20 mA·cm^–2^, with 5% NM2T showing superior performance (446 mV) compared to 2% NM2T (493 mV) at this higher current density. Further increase in MXene content to 10 wt % led to performance deterioration, although still maintaining better activity than the 1% NM2T composite, requiring 471 mV to achieve 10 mA·cm^–2^. The enhanced performance of the MXene-modified composites, particularly 5% NM2T, can be attributed to several factors: (I) The conductive Ti_3_C_2_T_*x*_ MXene likely facilitates electron transfer, with optimal electronic coupling achieved at 5 wt % loading; (II) MXene serves as conductive support, potentially preventing aggregation of NM2 particles and maintaining high active site accessibility; (III) The formation of beneficial interfaces between MXene and NM2 may contribute to the enhanced catalytic activity. The performance deterioration at higher MXene content (10 wt %) could be attributed to two causes: (I) Excessive MXene loading may block active sites, and (II) the inhomogeneous MXene-NM2 interface (as seen in SEM, Figure 2j) disrupts electronic coupling, offsetting the benefits of retained conductivity. The overall activity trend across all synthesized catalysts can be summarized in Figure 6c. Compared to the recently reported Ti_3_C_2_T_*x*_ MXene and 2D-based OER catalysts, the 5% NM2T composite exhibited comparable performance at 10 mA cm^–2^ (Table S3).

Detailed kinetic analysis and surface area characterization were conducted to elucidate the underlying mechanisms responsible for enhanced performance. Recently it has been shown that MXenes, including Ti_3_C_2_T_*x*_, reconstruct under anodic polarization under the formation of single-atom centers (SAC), reminiscent of single-atom catalysts.^56^ Hence, DFT calculation were carried out to give insight into the OER mechanism of the pure Ti_3_C_2_T_*x*_. Hence, the Ti_3_C_2_T_*x*_ surface is represented by a double-branched Ti_3_C_2_–SAC model with a spectator oxygen atom at the active center, as recently reported by Faridi et al.^57^ A surface model of the double-branched Ti_3_C_2_–SAC motif is shown in Figure S9a, while the corresponding free-energy diagram along the OER reaction coordinate is presented in Figure S9b. In this context, the OER is described by a mononuclear mechanism and a mononuclear-Walden mechanism (more information in the Supporting Information); the relevance of the latter for energy conversion processes at electrified interfaces has only recently been reported.^58,59^ Figure S9b illustrates that the SAC motif of Ti_3_C_2_T_*x*_ reveals activity for the OER. For both the mononuclear and mononuclear-Walden mechanisms, the OER activity is governed by the transition from the *OH to the *O intermediate, which is identified as the limiting reaction step in the context of the *G*_max_(U) descriptor at *U* = 1.40 V vs RHE (reversible hydrogen electrode). In terms of a NiMo covered Ti_3_C_2_T_*x*_, the reconstruction of the MXene under OER conditions may lead to a different NiMo structure compared to the pure NiMo due to the SAC formation, which is further discussed in the operando Raman.

For the composite materials, Tafel analysis (Figure 6d) revealed systematic improvements in reaction kinetics with MXene incorporation. The pristine NM2 exhibited a Tafel slope of 178 mV·dec^–1^, while the MXene-modified composites showed progressively lower values up to 5 wt % loading. The optimal 5% NM2T achieved a Tafel slope of 120 mV·dec^–1^, indicating more favorable reaction kinetics and faster electron transfer. This trend aligns with the enhanced OER activity observed in LSV measurements.

The ECSA measurements, determined through *C*_dl_ analysis, provided further insights into the performance enhancement mechanism, Table S4. Among the binary oxides, NM2 exhibited the highest *C*_dl_ (337 μF·cm^–2^) and ECSA (8.42 cm^2^), consistent with its superior OER activity. The integration of Ti_3_C_2_T_*x*_ MXene led to a dramatic increase in active surface area, with 5% NM2T showing the highest *C*_dl_ (775 μF·cm^–2^) and ECSA (19.37 cm^2^), representing a ∼2.3-fold increase compared to pristine NM2. The C_dl_ and ECSA values for all samples are listed in Table S4 in the Supporting Information.

### 3.2 Long-Term Stability Evaluation on Industrially Relevant Electrodes

Chronopotentiometry measurements were conducted on Ni felts (NF) at a constant current density of 10 mA·cm^–2^ for 24 h to assess the long-term operational stability (Figure 7a). During the initial stabilization period, most catalysts exhibited a conditioning phase during the first 4 h of operation, with the pristine NF showing the highest initial overpotential and significant stabilization requirements. Regarding steady-state performance, 5% NM2T demonstrated superior stability with the lowest overpotential (∼315 mV vs RHE) throughout the test. The overpotential remained remarkably stable after the initial conditioning period, with minimal degradation observed over the 24-h testing period. Stability exhibited a clear composition dependence. The binary NiMo oxides (NM1, NM2, NM3) showed intermediate stability, while MXene-modified composites generally exhibited better stability than their unmodified counterparts. Performance retention analysis revealed that all MXene-modified catalysts maintained stable performance throughout the extended operation. The optimal 5% NM2T showed negligible performance degradation, indicating robust structural stability. The enhanced stability of MXene-modified composites suggests effective integration of the conductive support.

**Figure 7:**
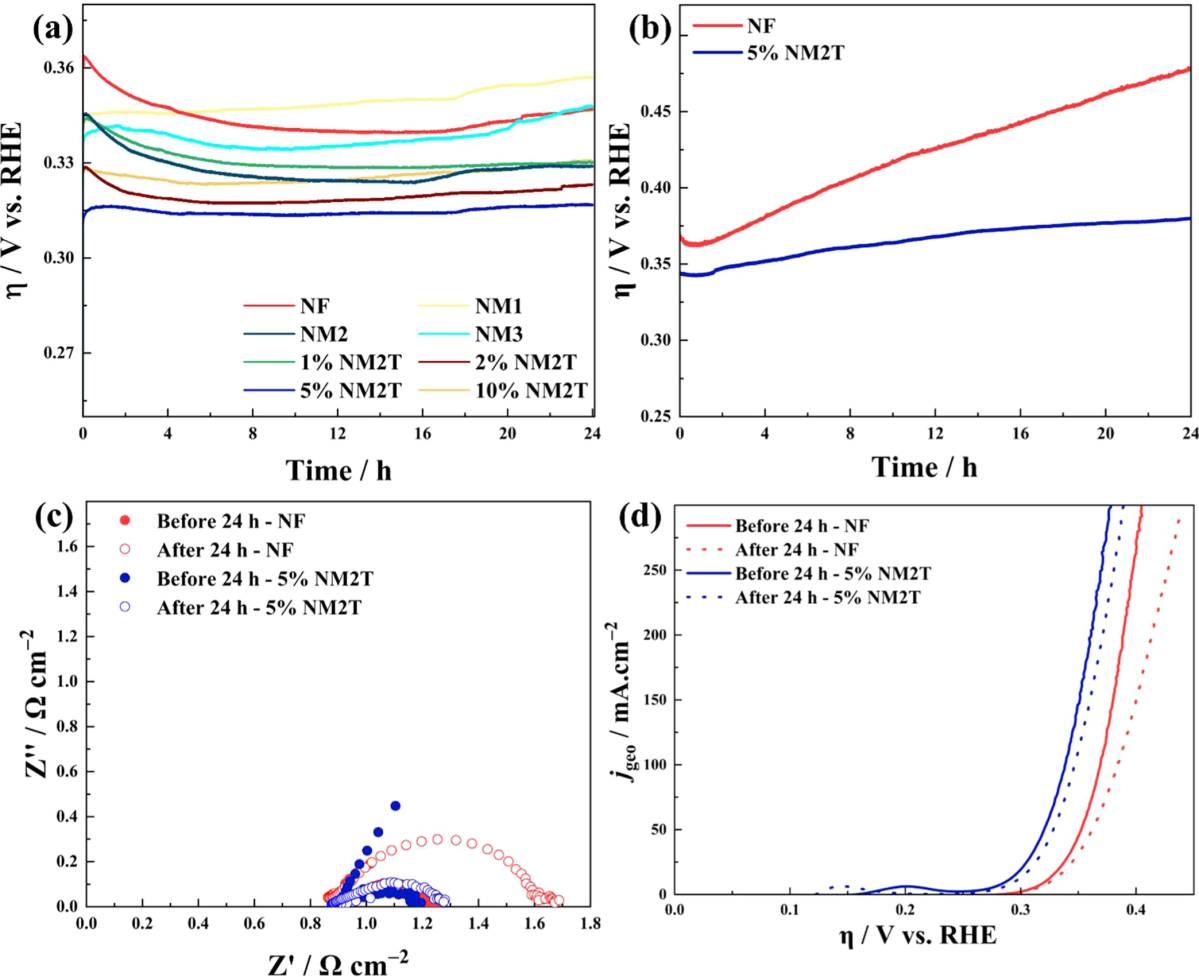
Chronopotentiometric behaviors of (a) the prepared samples at a constant current density of 10 mA·cm^–2^ and (b) 5% NM2T at a constant current density of 100 mA·cm^–2^ for 24 h on Ni Felts (NF). (c) Galvanostatic EIS at 100 mA•cm^–2^ before and after 24 h, and (d) LSV curves before and after 24 h for the 5% NM2T sample.

The 5% NM2T composite was further evaluated for high-current stability at 100 mA·cm^–2^. It exhibited an overpotential of 339 mV and maintained stable electrolysis performance, with a degradation rate of only 1.3 mV·h^–1^ over 24 h (Figure 7b). Additionally, the charge transfer resistance increased by about only 100 mΩ·cm^–2^, compared to bare nickel felt, which exhibited a resistance of approximately 380 mΩ·cm^–2^ after 24 h of electrolysis (Figure 7c). Furthermore, the 5% NM2T composite exhibits a 30 mV lower overpotential at 100 mA·cm^–2^ compared to the commercially available NiO powder catalysts (370 mV overpotential) and only 50 mV higher than NiFeOOH catalysts (Figure S10). LSV curves (Figure 7d) measured after the stability test revealed a minor increase of 10 mV at 100 mA·cm^–2^ after 24 h. All these results support that the 5% NM2T possesses high electrochemical stability and OER activity, making it a promising anode catalyst for alkaline water electrolysis.

### 3.3 Operando Raman Spectroscopy

The operando Raman spectroscopy reveals distinctive structural evolution patterns across the investigated materials during the oxygen evolution reaction. Operando Raman spectroscopy links real-time vibrational changes with potential-dependent current responses. Spectro-electrochemical measurements were conducted on NiO, NM2, 5% NM2T samples (optimum catalysts) and a bare Ti_3_C_2_T_*x*_, with Raman spectra collected at different electrical bias points to monitor surface bonding changes and catalyst phase evolution, as presented in Figure 8.

**Figure 8:**
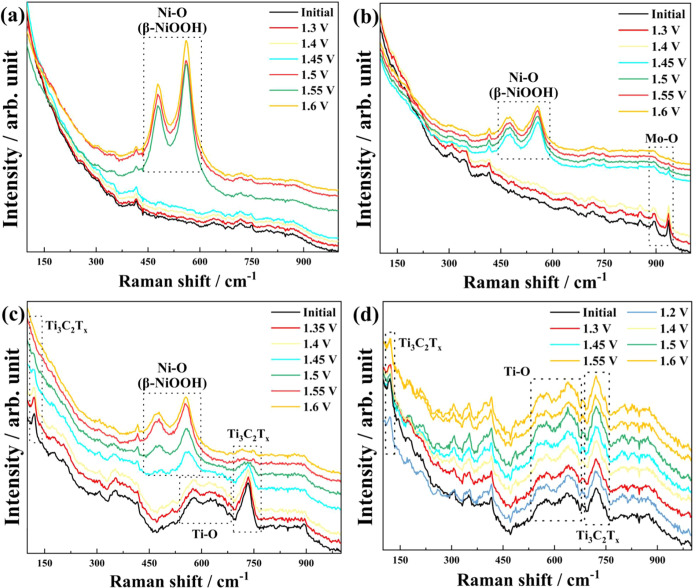
Operando Raman spectra of the (a) NiO, (b) NM2, (c) 5% NM2T, and (d) Ti_3_C_2_T_*x*_ composite during the OER with a potential sweep ranging from 1.3 to 1.6 V vs RHE.

The analysis of the spectra revealed a consistent peak at approximately 416 cm^–1^ across all samples and potentials, which was attributed to the gold electrode substrate. For pristine NiO (Figure 8a), in the initial spectrum without any applied bias, the vibration peaks related to the Ni–O are not clearly visible. Upon reaching 1.5 V (vs RHE), two characteristic peaks emerge at 479 and 560 cm^–1^, suggesting the formation of β-NiOOH as the more dominant nickel oxide hydroxide phase due to the higher intensity of the peak at 560 cm^–1^ compared to the 479 cm^–1^.^60,61^ These peaks correspond to Ni–O bending and stretching vibrations, respectively, and their positions agree with previous reports.^60,62,63^ The signals remained stable during operando measurements at 1.6 V (vs RHE), suggesting no further changes in the catalyst and indicating β-NiOOH as the active species for OER.

For the NiMoO_4_ sample (Figure 8b), the initial spectrum without applied potential showed primary bands at 894 and 936 cm^–1^, characteristic of the molybdate structure.^64^ The emergence of peaks at 477 and 558 cm^–1^ at 1.45 V (vs RHE) mirrors the β-NiOOH formation observed in NiO, suggesting that the nickel centers undergo similar oxidation processes but at lower potentials (compared to NiO), suggesting that the modified catalyst surface requires less energy for β-NiOOH formation and ultimately, the evolution of O_2_. In addition, the gradual decrease in Mo–O peak intensities (894 and 936 cm^–1^) with increasing potential indicates potential molybdenum leaching-out or partial amorphization of the molybdate framework during the OER, which is consistent with other work on pure NiMo materials for the OER.^39^

For the 5% NM2T sample (Figure 8c), the initial spectrum without applied potential showed primary bands at 576 and 733 cm^–1^, which are associated with the typical E_g(3)_ vibrations of Ti–O bonds and the vibrational modes of Ti_3_C_2_T_*x*_.^65^ Over the potential range from 1.35 to 1.6 V (vs RHE) both peaks seem to disappear, which could either suggest leaching of the Ti materials or the formation of a thick Ni oxide on the surface. Similarly to the NM2 sample, β-NiOOH is the major NIOOH formed at a potential of 1.45 V (vs RHE). However, the 477 and 558 cm^–1^ peak intensities differ between the 5% NM2T and NM2 samples which may account for the increase in OER activity of the 5% NM2T. At 1.45 vs RHE, the Raman spectra of the 5% NM2T sample indicates that more β-NiOOH is present. Bell and co-workers have previously shown that β-NiOOH is more OER active then the γ-NiOOH phase.^60^ Hence, as more β-NiOOH is present for the 5% NM2T sample, this is a clear reason for the increase in OER activity when compared to the pure NiMo. It is possible that the Ti_3_C_2_T_*x*_ MXene may promote the presence of the β-NiOOH phase due to the MXene acting as a support to preferentially make certain layered structures. In previous work, it was reported by Kaplan and co-workers that the presence of Ti_3_C_2_T_*x*_ during hydrothermal synthesis resulted in a Co layered double hydroxide formed while without Ti_3_C_2_T_*x*_, the pure Co was Co­(OH)_2_.^66^ In addition, these findings are supported by recent studies on Ni single-atom catalysts anchored on Ti_3_C_2_T_*x*_ MXene, which revealed similar high-valent Ni­(III/IV) transitions and strong Ni–O coordination environments during anodic operation, even in systems designed for organic oxidation rather than OER.^67–69^ While mechanistically different in application, these reports align with the observation of β-NiOOH formation in this study and underline the important role of the MXene support in stabilizing high-valent Ni species under anodic conditions. However, from the DFT calculations, Figures S1–S2, SAC sites are formed during pure Ti_3_C_2_T_*x*_ oxidation in the OER which could play a role in the restructuring of the Ni–Ti_3_C_2_T_*x*_ to a SAC like motif.

The evolution of β-NiOOH under anodic conditions was studied by analyzing the ratio of Raman band intensities at about 477 and 558 cm^–1^ (*I*_477_/*I*_558_), which both indicate β-NiOOH vibrational modes. As depicted in Figure S11, this ratio increased with potential across all samples, with the 5% NM2T composite showing the highest and most stable values (0.78–0.83). In contrast, NiO and NM2 had lower ratios, ranging from 0.53–0.54 and 0.56–0.70, respectively. The consistently higher *I*_477_/*I*_558_ ratio in the 5% NM2T system indicates more substantial formation of the β-NiOOH phase, supporting the view that the MXene support enhances the production and stabilization of catalytically active Ni­(III) species during the OER.

In the case of pristine Ti_3_C_2_T_*x*_ (Figure 8d), as the potential increased from 1.2 to 1.6 V (vs RHE), a gradual intensification of the Ti–O signal is observed, indicative of progressive surface oxidation of the MXene. This trend, though subtle, is reproducible and aligns with the known propensity of MXenes to undergo localized oxidation at the electrolyte interface under anodic bias.^70^ Critically, the Ti_3_C_2_T_*x*_-related peak at 733 cm^–1^ maintained its stability across all potentials, which confirms that the MXene backbone remains structurally intact under electrochemical conditions. No visible physical changes (e.g., dissolution, flaking) were observed on the electrode surface during testing. The selective oxidation of surface Ti species (evidenced by the Ti–O peak enhancement) aligns with MXene’s inherent low OER activity, which minimizes structural degradation under anodic bias.

### 3.4 Monitoring Molybdenum Etching with ICP-OES

To link molybdenum leaching with the formation of active phases and OER performance, the concentration of dissolved Mo in the electrolyte (post stability test for each sample) was assessed using ICP-OES (see Table S5). In pure NiMoO_4_ samples, Mo leaching significantly depended on the Ni/Mo stoichiometry. The Ni-rich NM3 and Mo-rich NM1 formulations displayed distinctly different leaching profiles: NM3 released 677 μg/L of Mo, whereas NM1 had minimal dissolution at 103 μg/L. Despite NM1′s lower Mo leaching, it did not achieve the target current density of 10 mA cm^–2^ within the tested potential range, likely due to an insufficient number of Ni sites for β-NiOOH formation. In contrast, NM3′s greater Mo leaching still yielded poor OER activity, as its Ni-dominated lattice hindered the essential cooperative interaction between Ni and Mo necessary for stabilizing active phases and facilitating charge transfer. Notably, NM2 displayed the highest Mo leaching at 941 μg/L, but its equimolar Ni/Mo stoichiometry assures that adequate residual Mo remains to stabilize the lattice and preserve Ni–Mo electronic interactions. This promotes β-NiOOH formation and OER activity, despite the considerable dissolution. These findings indicate that controlled Mo dissolution is vital for exposing Ni active sites while ensuring sufficient Mo retention to uphold structural integrity and electronic interactions.

The incorporation of Ti_3_C_2_T_*x*_ MXene alters Mo leaching behavior in a nonlinear manner. In MXene-containing composites, Mo leaching increased from 353 μg/L in 1% NM2T to 877 μg/L in 5% NM2T, before decreasing sharply to 197 μg/L in 10% NM2T. This trend parallels the OER activity profile, which peaked at 5% NM2T (η_10_ = 395 mV) and declined at higher loading levels. The dual functionality of MXene contributes to this optimum: (i) enhanced conductivity facilitates β-NiOOH formation, as evidenced by operando Raman, and (ii) the layered structure enables controlled Mo dissolution, generating defect-rich surfaces that promote active site exposure. Nickel leaching remained low across all samples (4–8 μg/L), and the persistence of the β-NiOOH Raman band at 558 cm^–1^ even at 1.6 V vs RHE confirms the structural stability of the Ni-based active phase.

A more detailed correlation between structural evolution and dissolution was established by comparing operando Raman spectroscopy with ICP-OES data for NM2 and 5% NM2T. In NM2, a 40–45% attenuation of the Mo–O vibrational bands at 894 and 936 cm^–1^ was observed under anodic polarization, aligning with its high Mo leaching value (941 μg/L). In contrast, 5% NM2T displayed only ∼5–10% Mo–O band attenuation, despite similarly high Mo dissolution. This suggests that MXene incorporation allows for a more controlled degradation pathway that preserves the catalyst’s framework while still exposing active Mo sites, contributing to the material’s superior OER performance.

To clarify the surface chemical alterations and structural integrity of the 5% NM2T catalyst during OER operation, XPS and EDS analyses were conducted before and after the 24-h chronopotentiometry test. The Ni 2p XPS spectrum (Figure S12) displayed a notable increase in Ni^3+^ features post-OER, indicating the formation and persistence of β-NiOOH, the active phase linked to oxygen evolution. On the other hand, the Mo 3d signal significantly reduced after electrolysis, indicating leaching of Mo from the catalyst surface, aligning with the ICP-OES results. EDS elemental mapping (Figure S13) further supported this, showing an apparent reduction in Mo distribution following the stability test, alongside an evident increase in Ti signal intensity. This relative Ti enrichment is linked to Mo depletion at the surface, revealing more of the underlying MXene support. These observations suggest that controlled Mo leaching exposes additional active Ni sites and shows the conductive MXene framework, potentially enhancing electron transport further.

## 4 Conclusions

This study systematically investigated how the Ni/Mo ratio and Ti_3_C_2_T_*x*_ MXene content affect the OER performance of NiMoO_4_-based catalysts in alkaline environments. NiMoO_4_ with varying Ni/Mo ratios and NiMoO_4_/Ti_3_C_2_T_*x*_ MXene composites containing 1–10% Ti_3_C_2_T_*x*_ were successfully prepared using a simple hydrothermal synthesis method. Comprehensive characterization using SEM, XRD, XPS, and Raman spectroscopy confirmed the successful synthesis of NiMoO_4_ and integration onto the MXene surface. It also highlighted the critical role of the Ni/Mo ratio in determining the morphology and performance of the catalyst. Electrochemical testing of the binary metal oxides has shown that an equimolar ratio of nickel to molybdenum (NM2) provides the best catalytic performance. This composition achieves an overpotential of 491 mV at a current density of 10 mA·cm^–2^, significantly outperforming molybdenum-rich (NM1) and nickel-rich (NM3) compositions.

Furthermore, the optimal amount of Ti_3_C_2_T_*x*_ MXene additive was found to be 5%, which exhibited a notably lower overpotential of 395 mV at 10 mA·cm^–2^, representing an improvement over the pristine NiMoO_4_ catalyst. This improvement can be attributed to the synergistic effects of increased electrical conductivity, increased amount of β-NiOOH during OER and a 2.3-fold increase in electrochemically active surface area (19.37 cm^2^ compared to 8.42 cm^2^ for pristine NiMoO_4_). ζ-Potential measurements revealed that increasing MXene content progressively shifted the surface charge toward more negative values (−3.07 mV → −7.30 mV), reflecting enhanced interfacial interactions and MXene character at optimal loadings. Operando Raman spectroscopy demonstrated that while NiO formed β-NiOOH active species at 1.5 V vs RHE, NM2 achieved this transformation at a lower potential of 1.45 V and exhibited a greater amount of β-NiOOH when compared to the pure Ni and NiMo samples. The 5% NM2T composite also demonstrated outstanding stability during 24-h chronopotentiometry testing on industrially relevant metal gas diffusion Ni fiber felts, maintaining a stable overpotential of approximately 288 mV at 10 mA cm^–2^ while reaching an overpotential of ca. 339 mV at 100 mA cm^–2^. ICP-OES leaching studies revealed a volcano-type dependence of Mo dissolution and MXene loading, peaking at 5 wt % (877 μg/L) before declining at 10 wt % (197 μg/L). This closely parallels the trend in electrocatalytic activity, while Ni dissolution remained low (<8 μg/L). Ex situ XPS and EDS mapping following 24 h of chronopotentiometry demonstrated increased Ni^3+^ content, depletion of Mo, and a relative enrichment of Ti on the 5% NM2T surface. This suggests that controlled Mo leaching uncovers additional active Ni sites and reinforces the conductive MXene network.

These results reveal a distinct structure–activity relationship: an optimal Ni/Mo ratio guarantees adequate Mo retention for lattice stability, while precise MXene integration improves conductivity, surface charge modulation, and regulated Mo leaching to enhance β-NiOOH exposure. These findings are further supported by DFT calculations, which confirm that the Ti_3_C_2_T_*x*_ MXene surface is catalytically active for OER via a mononuclear mechanism, with the *OH to *O transition as the potential-determining step, correlating well with the enhanced Ni^3+^ formation and β-NiOOH stabilization observed experimentally. The 5% NM2T composite demonstrates exceptional OER performance and long-term resilience, offering a solid design approach for MXene-supported electrocatalysts in sustainable hydrogen technologies.

## Supplementary Material



## Notes

Computational details; Gibbs free energy and CHE model; reaction mechanisms of mononuclear and mononuclear-Walden pathways; free-energy changes and descriptor analysis; EIS resistance data; High-resolution XPS and EDS spectra; XRD patterns of MAX phase, MXene, and composites; ζ-potential measurements; free-energy diagrams of OER; LSV curves; Operando Raman data; poststability test XPS and EDS mapping; comparison table of OER catalysts; *C*_dl_ and ECSA data; metal leaching and overpotential data (PDF)
