# Supplementary material for: Compositional Engineering of Ti3C2T x MXene-NiMoO4 Hybrid Nanostructures for Enhanced Electrocatalytic Water Oxidation
Source: ACS Appl Energy Mater. 2025 Jul 25;8(15):11313–28. doi: 10.1021/acsaem.5c01467 (PMC12344690; doi:10.1021/acsaem.5c01467)
Supplement: Supplementary file 1 [file ae5c01467_si_001.pdf]

Supporting Information

Compositional Engineering of $Ti_3C_2T_x$ MXene-$NiMoO_4$ Hybrid Nanostructures for Enhanced Electrocatalytic Water Oxidation

Saeed Sajjadi[1,2], Thorsten Schultz[3,4], Danielle A. Douglas-Henry[5], Karuppasamy Dharmaraj[1], Aline Alencar Emerenciano[1], Can Kaplan[1], Noel Marks[6], Kai S. Exner[6,7,8], Valeria Nicolosi[5], Norbert Koch[3,4] and Michelle P. Browne[1]*

1. Helmholtz Young Investigator Group Electrocatalysis: Synthesis to Devices, Helmholtz-Zentrum Berlin für Materialien und Energie GmbH, Albert-Einstein-Str. 15, 12489 Berlin, Germany.
2. Centre for Functional and Surface Functionalized Glass, Alexander Dubček University of Trenčín, Trenčín, 911 50, Slovakia
3. Helmholtz-Zentrum Berlin für Materialien und Energie GmbH, Berlin, 14109, Germany.
4. Institut für Physik & CSMB, Humboldt-Universität zu Berlin, Berlin, 12489, Germany.
5. School of Chemistry, CRANN and AMBER Research Centres, Trinity College Dublin, College Green, Dublin D02 PN40, Ireland.
6. University Duisburg-Essen, Faculty of Chemistry, Theoretical Catalysis and Electrochemistry, Universitätsstraße 5, 45141 Essen, Germany
7. Cluster of Excellence RESOLV, Bochum, Germany
8. Center for Nanointegration (CENIDE) Duisburg-Essen, Duisburg, Germany

*Email: Michelle.browne@helmholtz-berlin.de

S1 Computational details

Electronic structure calculations were carried out within the density functional theory (DFT) framework. The Vienna *Ab initio* Simulation package (VASP) [1–4], with the Perdew-Burke-Ernzerhof (PBE) [5] exchange correlation functional and the D4 [6] correction scheme were used to account for van der Waals interactions. The effects of the core electrons on the valence electron density were considered with the projector augmented wave (PAW) [7] approach. Solvation effects of water with a permittivity of 78.4 were taken into consideration using the VASPsol [8–10] extension. The kinetic cutoff energy for the plane wave basis set representing the valence electron density was set to 440 eV. The convergence criteria for the total energy and the maximum force threshold were set to 10^{-6} eV and 0.01 eV Å^{-1}, respectively. The reciprocal space was divided into a 5 x 5 x 1 Γ-centered k-point mesh.

To ensure physical isolation of the MXene layers along the direction perpendicular to the surface, a vacuum slab with a thickness of about 20 Å was included in the model, which prevented periodic image interaction. The reconstructed Ti_3C_2-SAC (single-atom-center) surface is modelled by taking the fully oxygen-covered Ti_3C_2 surface and introducing a further oxygen atom to lift one metal atom out of the basal plane. In addition, a spectator oxygen adsorbate is introduced at the active site, as shown in Fig. 9a in the main text.

S2 Gibbs Free Energies and Computational Hydrogen Electrode Model

The zero-point energy, E_{ZPE}, and the entropic contribution, TS, were determined by frequency calculations using the DFT framework. Normally the entropic contribution consists of the sum of all translational, rotational and vibrational frequencies; however, for adsorbed species, it is justified to neglect the translational and rotational parts [11,12]. For the calculation of the zero-point energy and vibrational entropy, we use equations (1) and (2), respectively:

$$E_{ZPE} = \frac{1}{2}\sum_i h \cdot \nu_i \tag{1}$$

$$T \cdot S = k_B \cdot T \sum_i^n \left[\frac{\frac{h \cdot \nu_i}{k_B \cdot T}}{\exp\left(\frac{h \cdot \nu_i}{k_B \cdot T}\right) - 1} - \ln\left(1 - \exp\left(\frac{-h \cdot \nu_i}{k_B \cdot T}\right)\right) \right] \tag{2}$$

In the above equation, k_B, h, v_i, n, and T denote the Boltzmann constant, Planck's constant, frequency of vibration, total number of frequencies, and temperature in Kelvin. Zero-point energy and entropic corrections at T = 298.15 K are needed to derive free energies according to the following relation:

$$G = E_{DFT} + E_{ZPE} - T \cdot S \tag{3}$$

The main text discusses the free-energy changes, ΔG, used to model the OER on the double-branched Ti_3C_2-SAC motif. These free-energy changes are determined using the computational hydrogen electrode (CHE) approach. For this purpose, the free energy of water at 298.15 K and 0.035 bar, as well as the free energy of a gaseous hydrogen molecule, were calculated, as these serve as reference states in the computational hydrogen electrode (CHE) model. The free-energy changes obtained from the CHE approach correspond to U = 0 V vs. SHE (standard hydrogen electrode) and pH = 0; this reference state is equivalent to U = 0 V vs. RHE (reversible hydrogen electrode). The RHE scale is used for discussing the energetics in the main text.

S3 Reaction Mechanism of the Oxygen Evolution Reaction

The OER consists of four proton-coupled electron transfer steps, in which different intermediates are formed in each reaction step. Two different mechanistic pathways are considered in this work: the mononuclear and the mononuclear-Walden mechanisms. The elementary steps of the mononuclear mechanism [13] is given by equations (4) – (7), while Fig. S1 provides a sketch of the elementary steps.

$$* + \mathrm{H_2O} \rightarrow * - \mathrm{OH} + \mathrm{H^+} + \mathrm{e^-},\ \Delta G_1 \tag{4}$$
$$* - \mathrm{OH} \rightarrow * - \mathrm{O} + \mathrm{H^+} + \mathrm{e^-},\ \Delta G_2 \tag{5}$$
$$* - \mathrm{O} + \mathrm{H_2O} \rightarrow * - \mathrm{OOH} + \mathrm{H^+} + \mathrm{e^-},\ \Delta G_3 \tag{6}$$
$$* - \mathrm{OOH} \rightarrow * + \mathrm{H^+} + \mathrm{e^-} + \mathrm{O_2},\ \Delta G_4 \tag{7}$$

It is important to note that the calculation of the O_2 molecule in Equation (7) is error-prone due to the inadequate description of the oxygen–oxygen double bond in plane-wave DFT calculations. To address this issue, gas-phase error corrections [14] are applied, and the free-energy change ΔG_4 is obtained using the following relation:

$$\Delta G_4 = 1.23\ \mathrm{eV} \cdot 4 - \sum_{i}^{3} \Delta G_i \tag{8}$$

The elementary steps for the mononuclear-Walden mechanism are given by equations (9) – (12) [13]:

$$* - \mathrm{OH} \rightarrow * - \mathrm{O} + \mathrm{H}^+ + \mathrm{e}^-, \ \Delta G_5 \quad (9)$$

$$* - \mathrm{O} + \mathrm{H_2O} \rightarrow * - \mathrm{OOH} + \mathrm{H}^+ + \mathrm{e}^-, \ \Delta G_6 \quad (10)$$

$$* - \mathrm{OOH} \rightarrow * - \mathrm{OO} + \mathrm{H}^+ + \mathrm{e}^-, \ \Delta G_7 \quad (11)$$

$$* - \mathrm{OO} + \mathrm{H_2O} \rightarrow * - \mathrm{OH} + \mathrm{H}^+ + \mathrm{e}^- + \mathrm{O_2}, \ \Delta G_8 \quad (12)$$

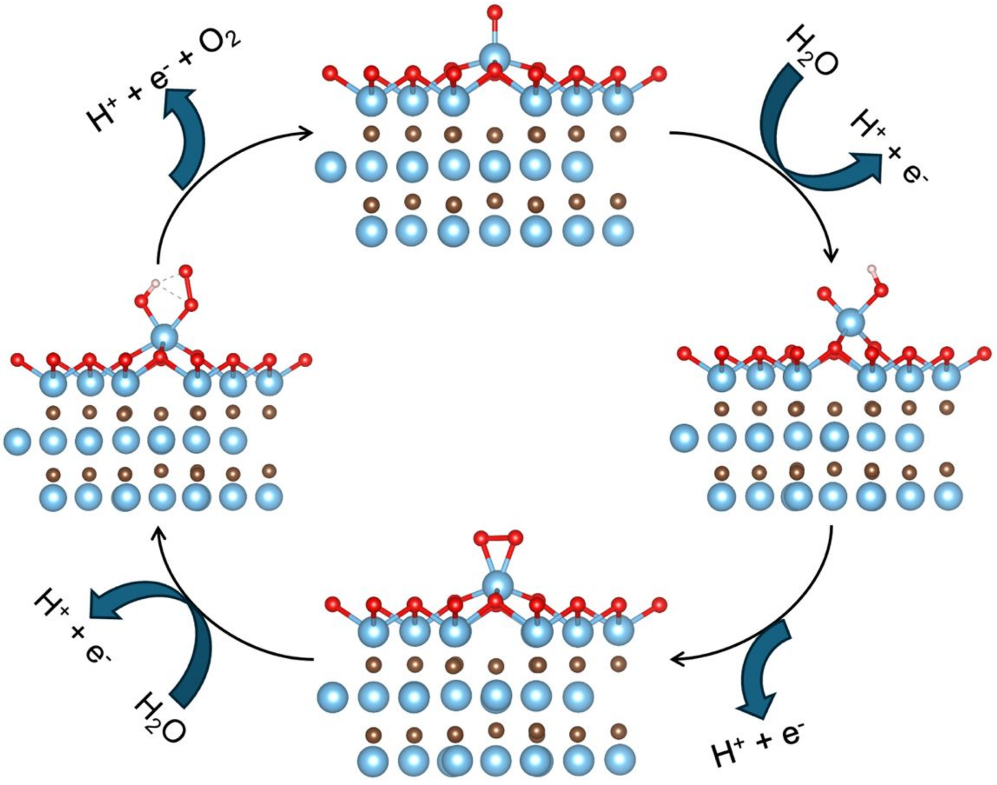

Fig. S1 Mechanistic description of the OER mononuclear mechanism with the preferred intermediate structures of the double-branched Ti_3C_2-SAC motif.

The concept of gas-phase error corrections [14] is also applied to the free-energy change ΔG_8 associated with the mononuclear-Walden mechanism. The catalytic cycle of the mononuclear-Walden pathway on the double-branched Ti_3C_2-SAC motif is illustrated in Fig. S2.

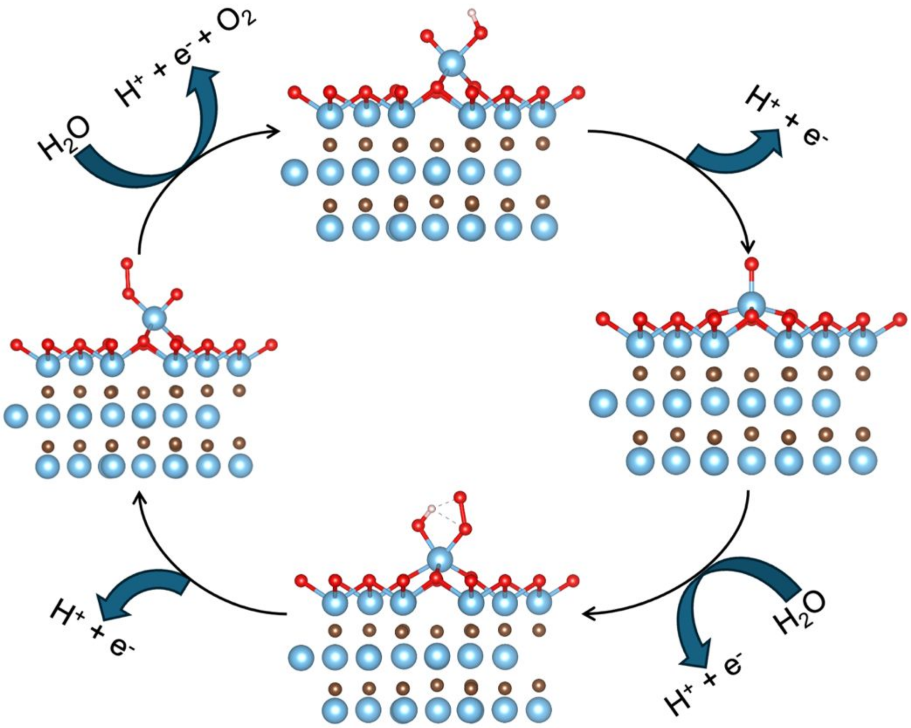

Fig. S2 Mechanistic description of the OER mononuclear-Walden mechanism with the preferred intermediate structures of the double-branched Ti_3C_2-SAC motif.

S4 Computed Free-Energy Changes

The computed free-energy changes of the elementary steps of equations (1) – (8) are compiled in **Table S1**.

Table S1 Free-energy changes of the mononuclear and mononuclear-Walden mechanisms. All values are given in eV.

ΔG_1	ΔG_2	ΔG_3	ΔG_4	ΔG_5	ΔG_6	ΔG_7	ΔG_8
1.16	1.53	1.22	1.02	1.53	1.22	0.84	1.33

S5 Descriptor-based analysis

To investigate the reaction kinetics of the different OER mechanisms, the thermodynamic free-energy diagram along the reaction coordinate (cf. Fig. 9b in the main text) is used to determine the activity descriptor $G_{max}(U)$, defined as the largest free-energy span between reaction intermediates at a given electrode potential [15]:

$$G_{\max} = \max_{i<j}\left(\Delta G_j - \Delta G_i\right) \tag{8}$$

This approach is justified because previous work by one of the authors showed that the descriptor $G_{max}(U)$ scales with the rate-determining transition state, thus allowing predictions of the kinetics using thermodynamic concepts [15]:

$$G_{max} = G^{\#}_{rds} - const. \tag{9}$$

Table S2 Uncompensated Solution Resistance (R_u) Values Determined by EIS at −0.2 V for All Samples

**Sample**	**R_u (Ω)**
NiO	13.33 ± 0.4
MoO_3	14.16 ± 0.16
NM1	14.34 ± 0.27
NM2	13.24 ± 0.46
NM3	14.38 ± 0.77
$Ti_3C_2T_x$	5.46 ± 0.19
1% NM2T	13.25 ± 0.52
2% NM2T	14.60 ± 1.25
5% NM2T	13.21 ± 0.20
10% NM2T	13.32 ± 0.23

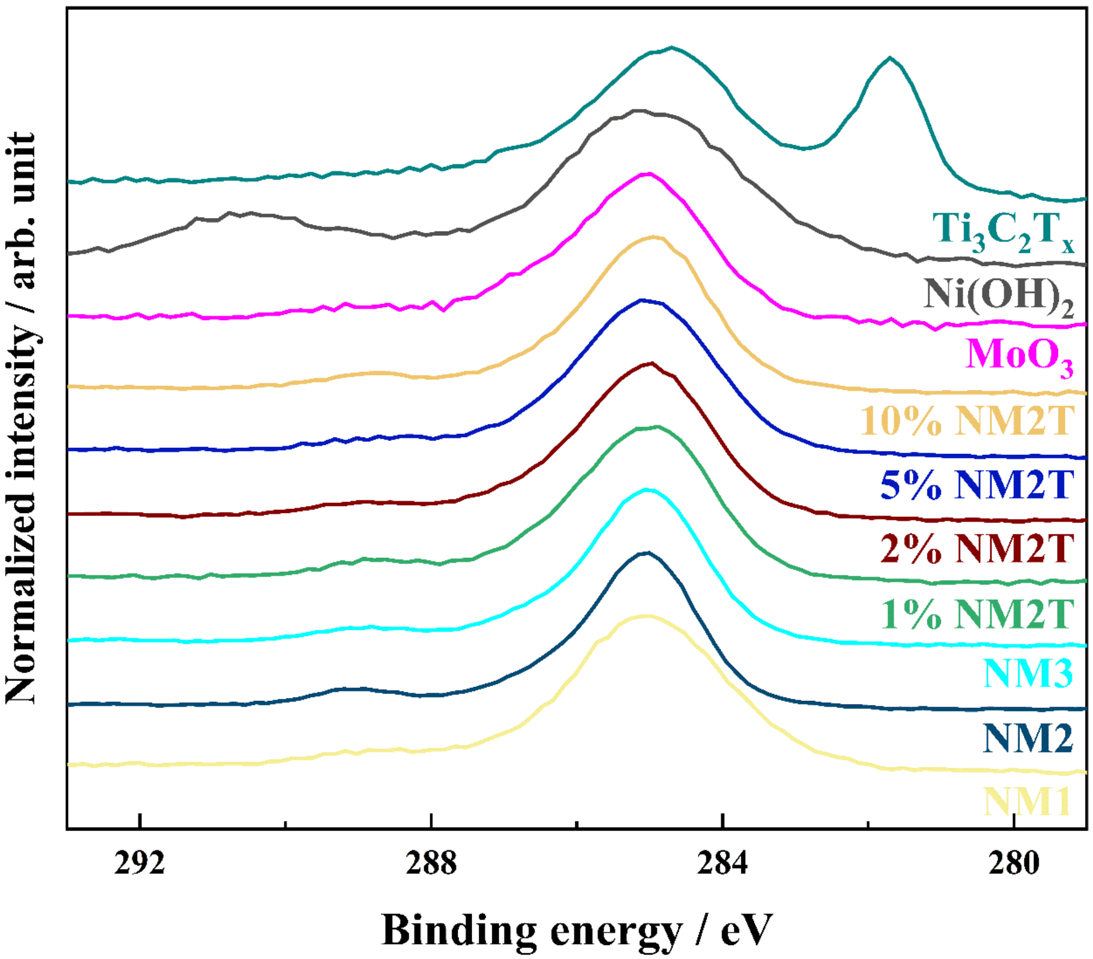

Fig. S3 High resolution C 1s core level.

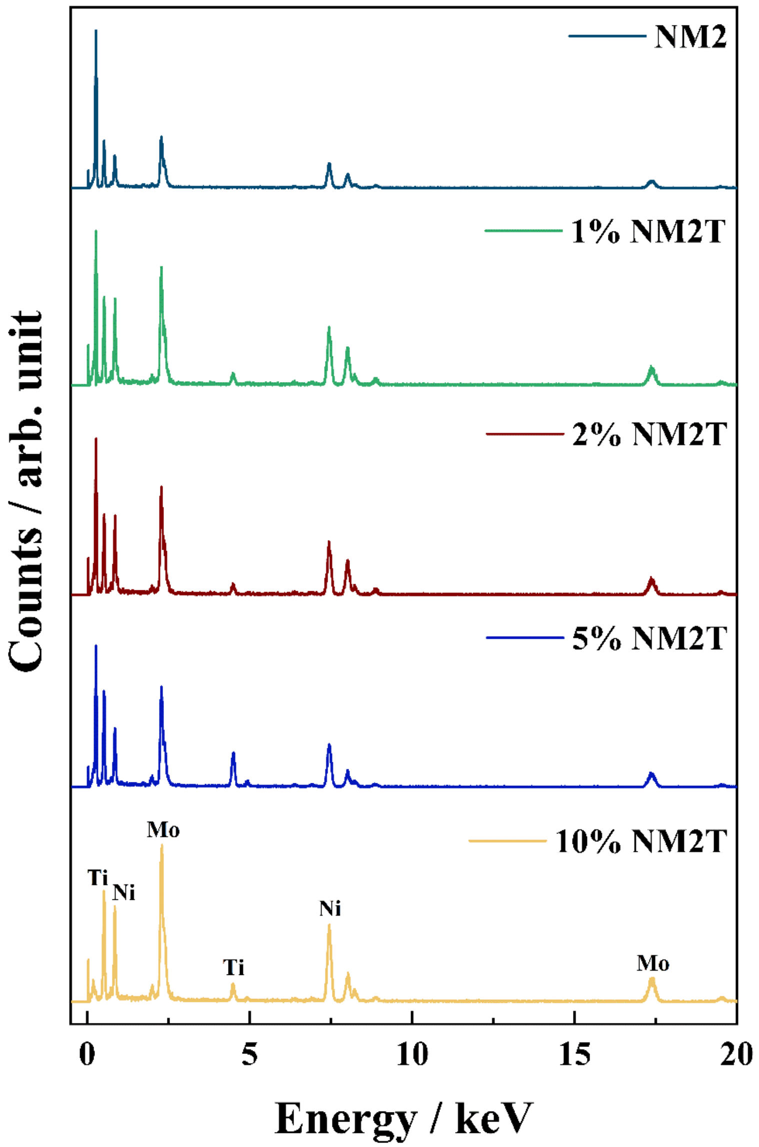

Fig. S4 EDS Spectrum of pure NM2, 1% NM2T, 2% NM2T, 5% NM2T and 10% NM2T.

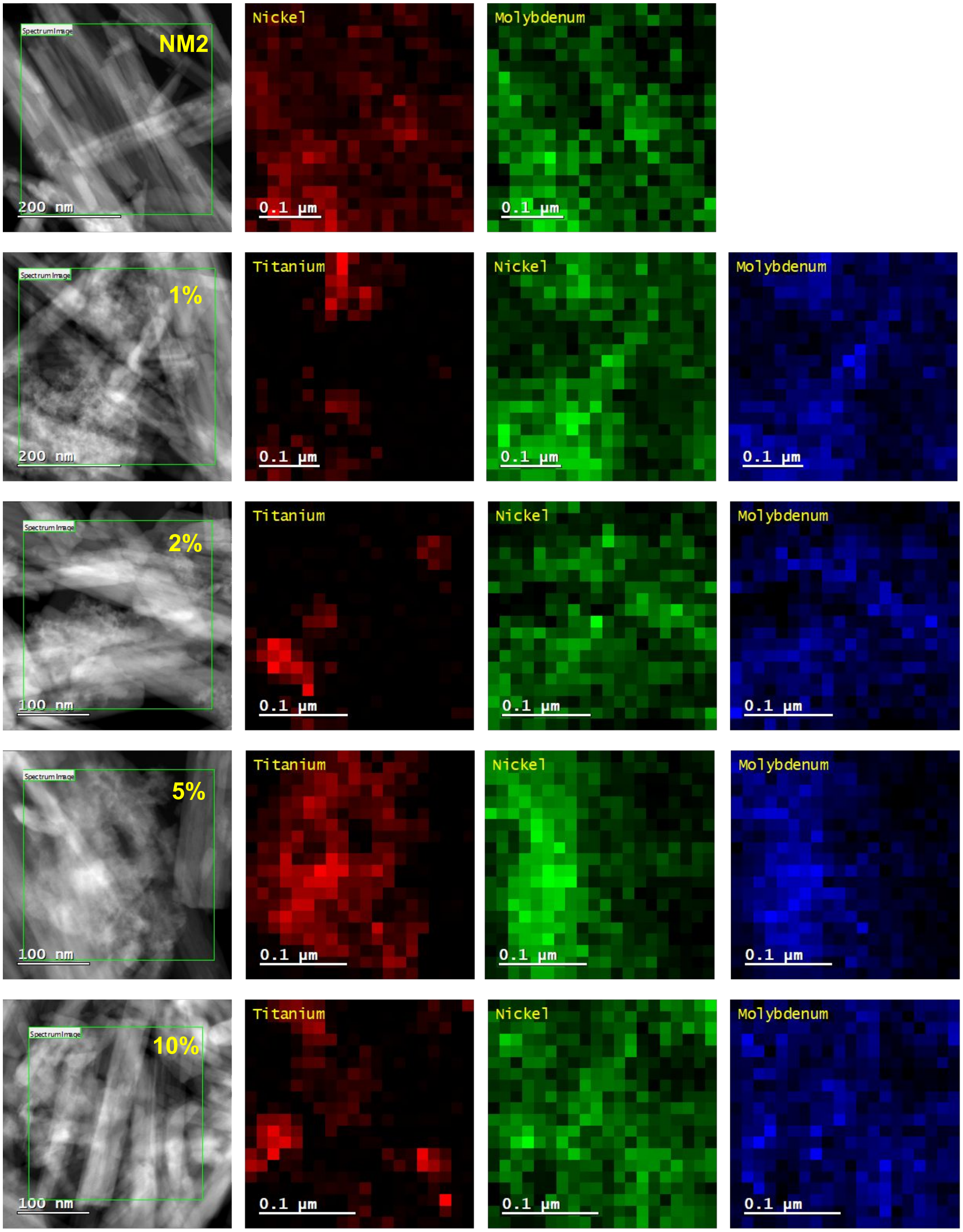

Fig S5. EDS map of pure NM2, 1% NM2T, 2% NM2T, 5% NM2T and 10% NM2T.

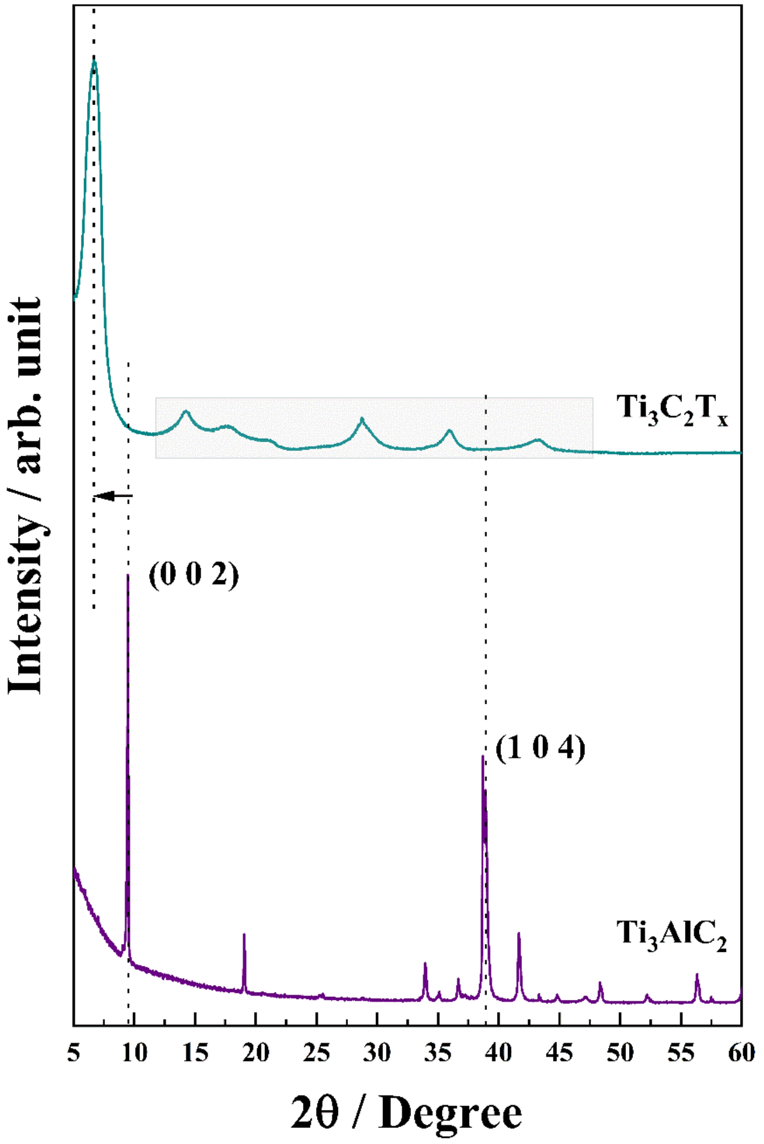

Fig. S6 XRD patterns of Ti_3AlC_2 MAX phase and $Ti_3C_2T_x$ MXene.

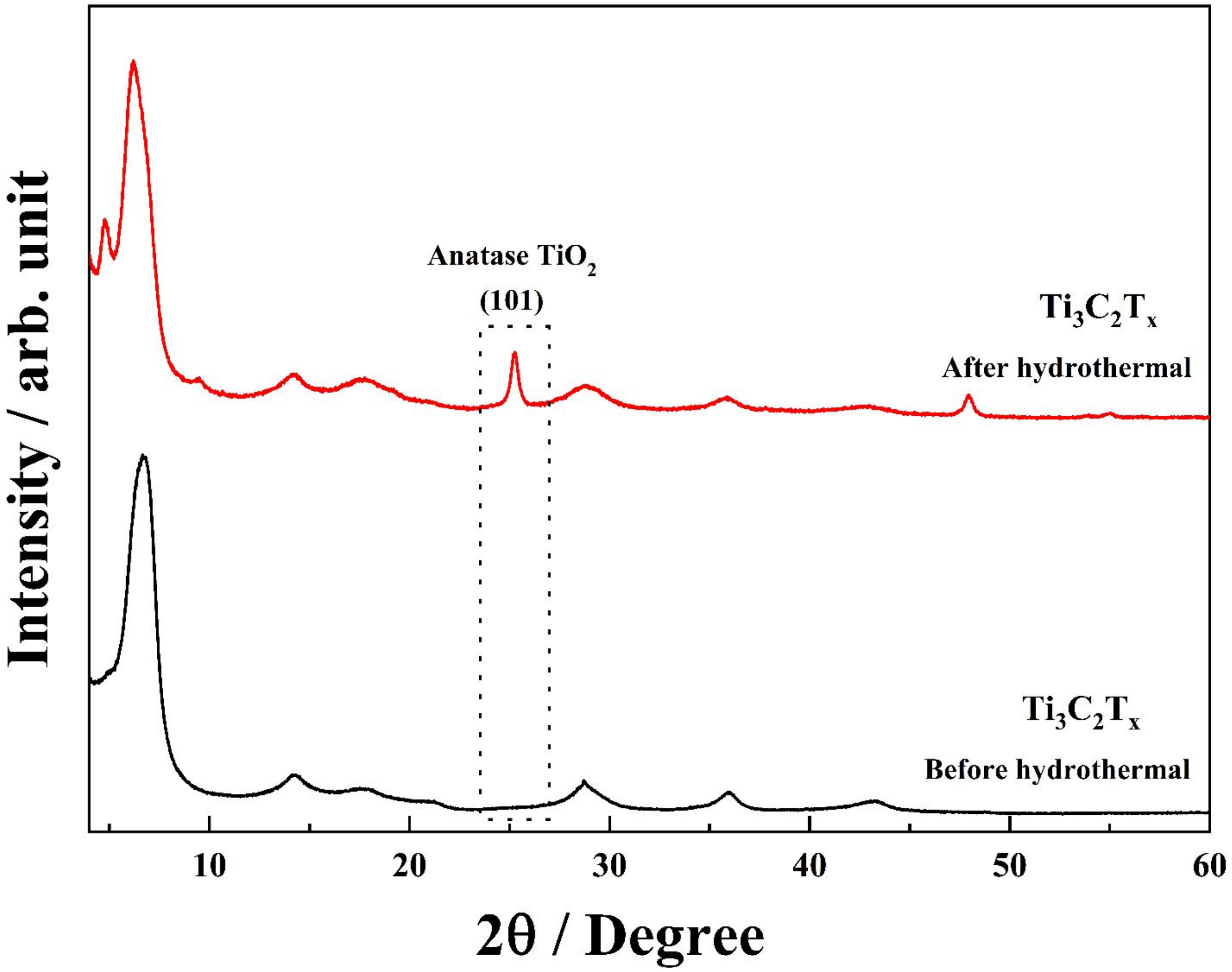

Fig. S7 XRD patterns of $Ti_3C_2T_x$ MXene and hydrothermally treated $Ti_3C_2T_x$ MXene.

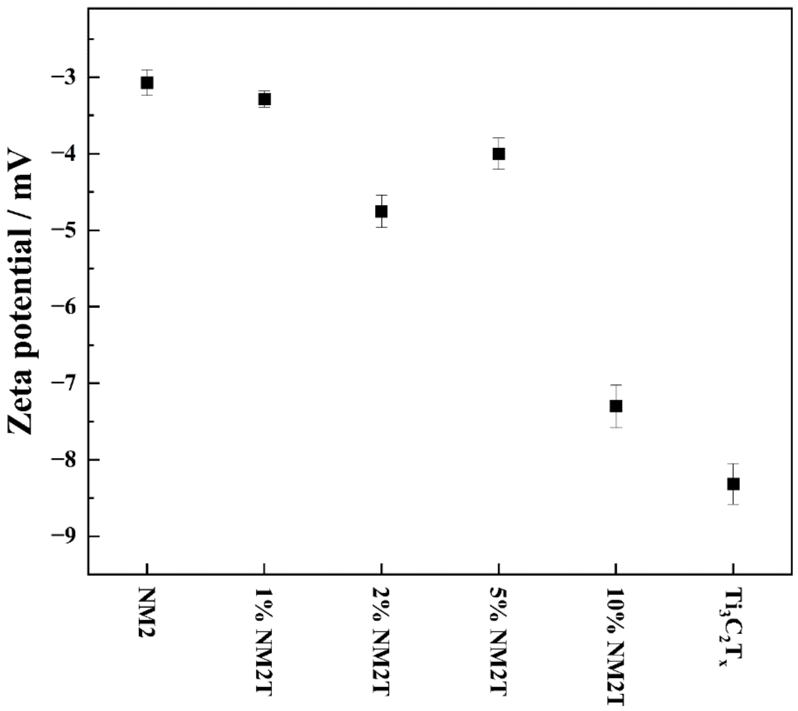

Fig. S8 Zeta potentials of pure NiMo, Ti3C2Tx and composites 1%T NM2, 2%T NM2, 5%T NM2 and 10%T NM2.

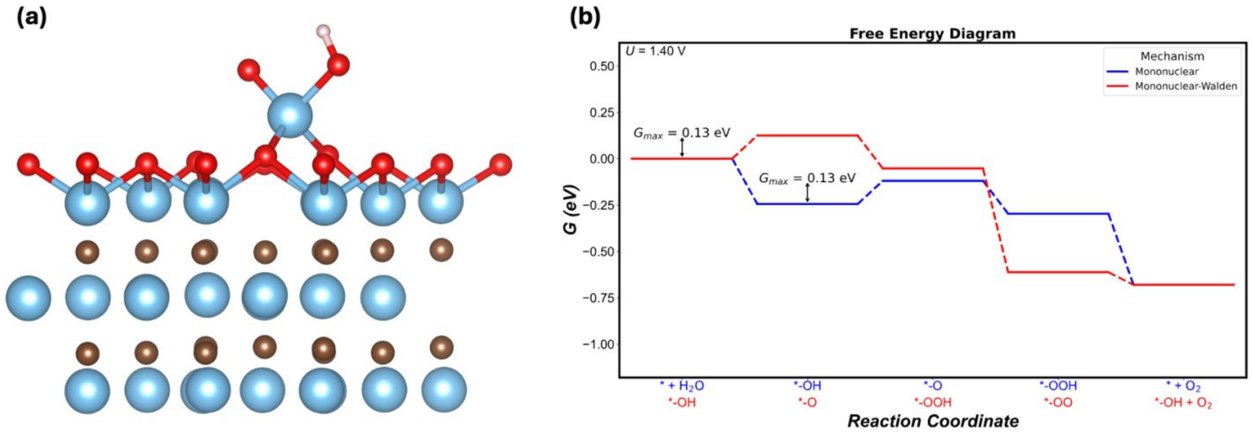

Fig. S9 (a) Double-branched Ti_3C_2-SAC motif used for studying the oxygen evolution reaction (OER). Blue, brown, and red atoms denote titanium, carbon, and oxygen atoms. While the *O adsorbate at the out-of-plane metal atom (SAC site) is a spectator atom, the elementary steps of the OER are modeled on the right branch of the SAC site. b) Free-energy diagram of the OER on Ti_3C_2-SAC at U = 1.40 V vs. RHE (reversible hydrogen electrode). An arrow indicates the descriptor $G_{max}(U)$.

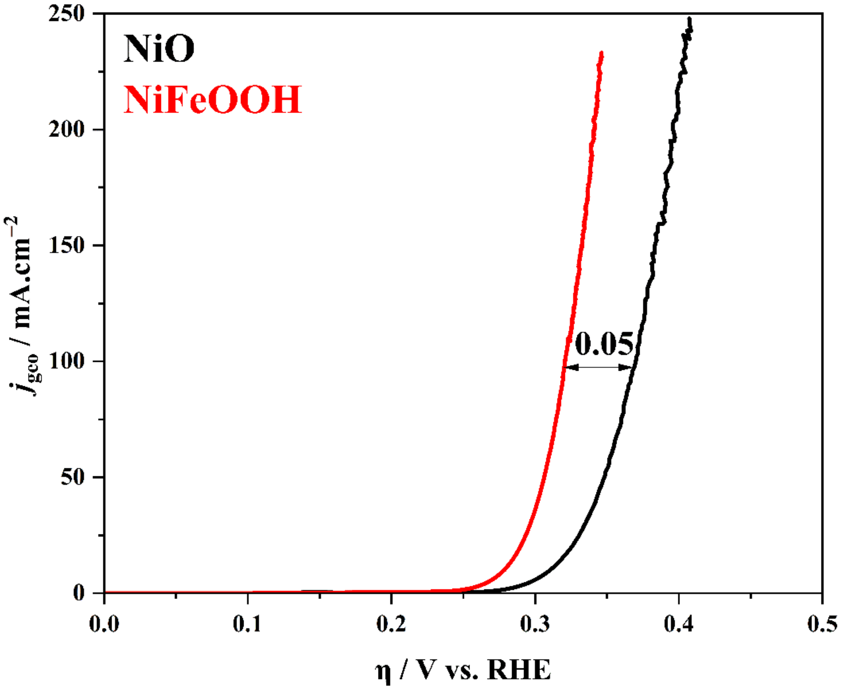

Fig. S10 LSV curves of commercial powder NiO (1 mg cm^{-2}) and electrodeposited NiFeOOH catalyst.

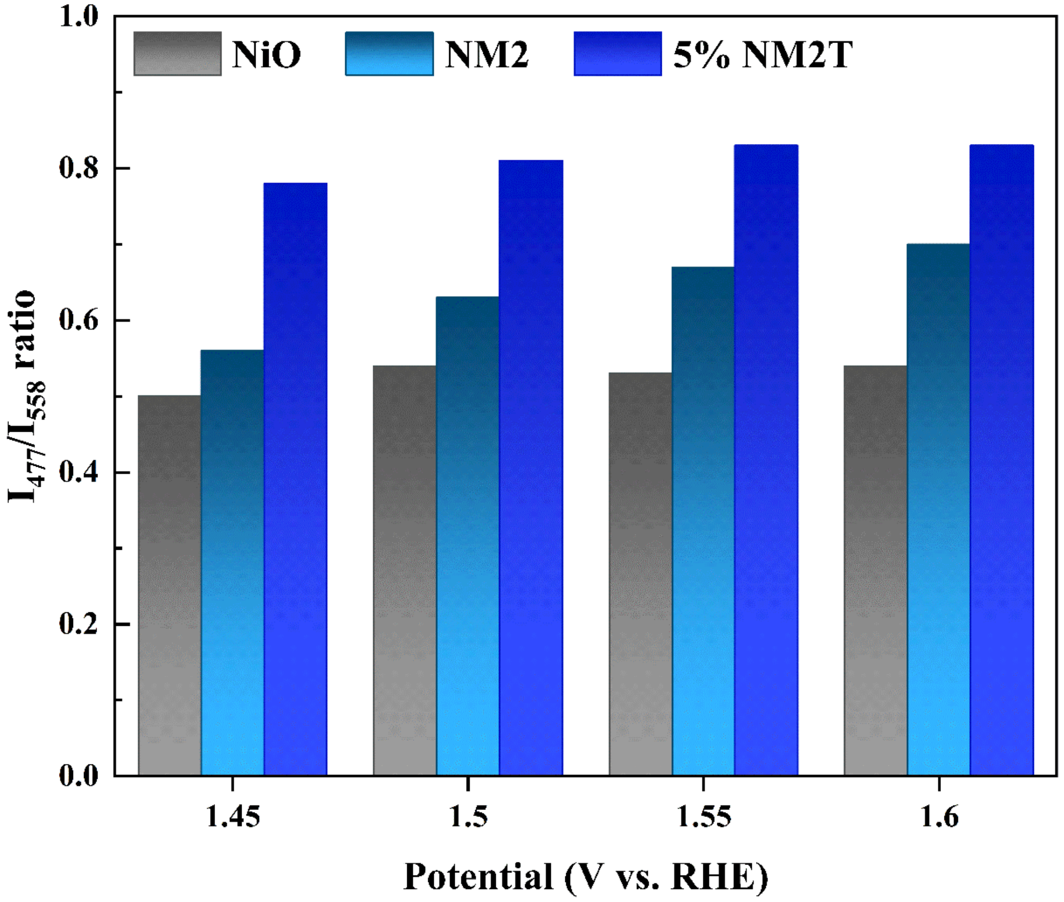

Fig. S11 Operando Raman-based intensity ratio of the β-NiOOH vibrational bands at 477 cm^{-1} and 558 cm^{-1} (I_{477}/I_{558}) as a function of applied potential for NiO, NM2, and 5% NM2T samples.

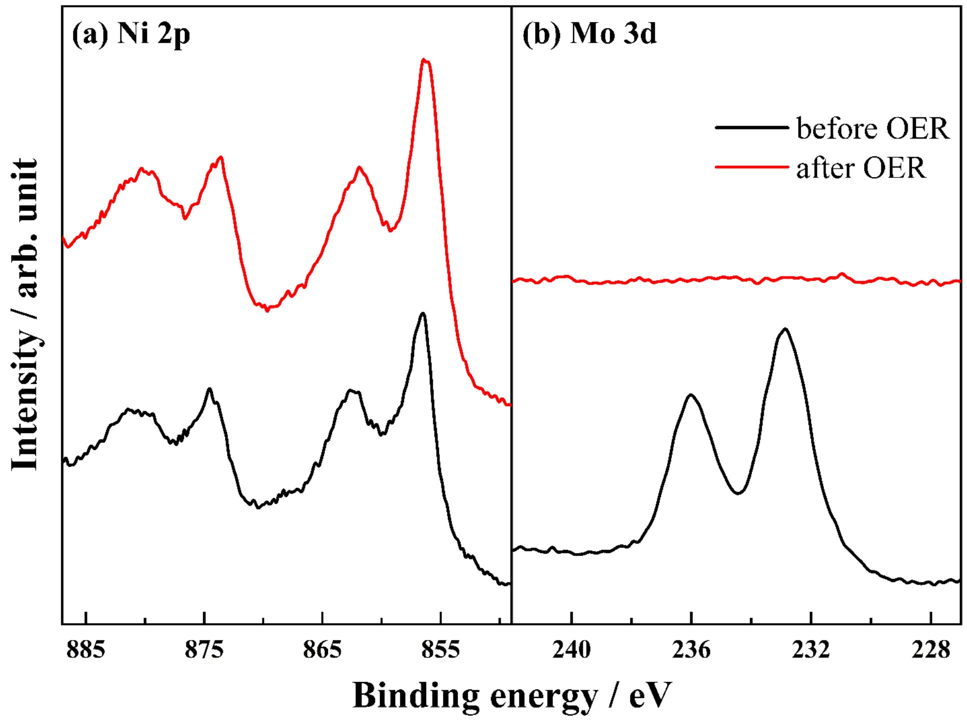

Fig. S12 High resolution (a) Ni 2p and (b) Mo 3d core level for 5% NM2T sample before and after 24 h chronopotentiometry test.

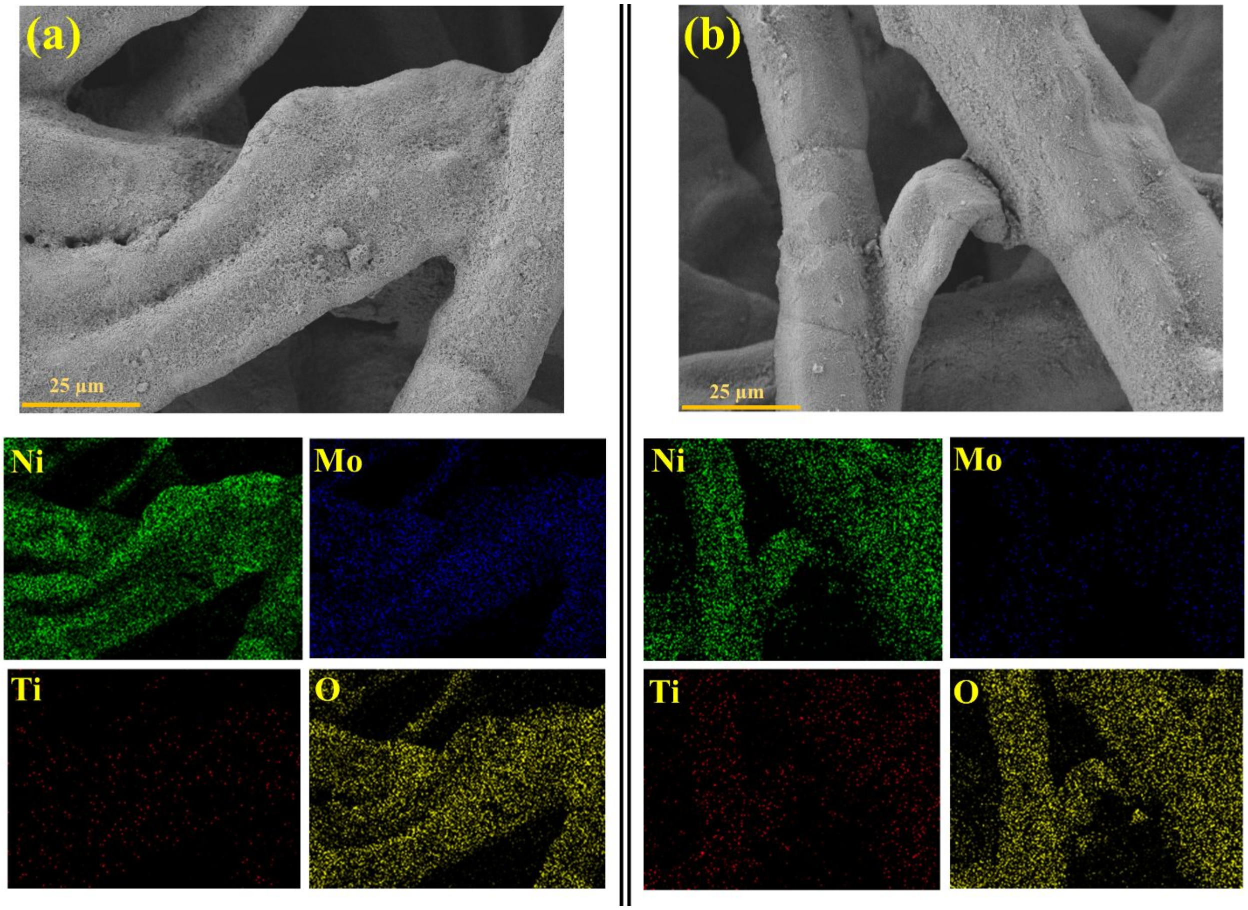

Fig. S13 EDS elemental mapping for 5% NM2T (a) before and (b) after 24 h chronopotentiometry test.

Table S3 Comparison of OER overpotentials of recently reported Ti_3C_2 MXene-based OER catalysts and this work.

Catalysts	Overpotentials	References
**Ti_3C_2 MXene-$NiMoO_4$**	0.39 V at 10 $mA\cdot cm^{-2}$	This work
**Ti_3C_2 MXene-Co-doped $Ni(OH)_2$**	0.31 V at 10 $mA\cdot cm^{-2}$	[16]
**Ti_3C_2 MXene-NiFeS**	0.29 V at 20 $mA\cdot cm^{-2}$	[17]
**Ti_3C_2 MXene-NiFe-LDH**	0.33 V at 10 $mA\cdot cm^{-2}$	[18]
**Co_4MnFe_3/Ti_3C_2 MXene**	0.26 V at 10 $mA\cdot cm^{-2}$	[19]
**FeOOH/ Ti_3C_2 MXene**	0.4 V at 10 $mA\cdot cm^{-2}$	[20]
**$FeOOH/Ti_3C_2$**	0.33 V at 10 $mA\cdot cm^{-2}$	[21]
**NiFeRh-LDH**	0.20 V at 10 $mA\cdot cm^{-2}$	[22]
**NiCo-LDH**	0.42 V at 10 $mA\cdot cm^{-2}$	[23]
**$NiCo_2S_4$@graphene**	0.47 V at 10 $mA\cdot cm^{-2}$	[24]

Table S4 C_{dl} and ECSA values for all of the prepared samples.

Sample	C_{dl} [μF·cm^{-2}]	ECSA [cm^2]
$Ti_3C_2T_x$	108	2.7
NiO	306	7.65
MoO_3	218	5.45
NM1	267	6.67
NM2	337	8.42
NM3	308	7.7
1% NM2T	689	17.22
2% NM2T	726	18.15
5% NM2T	775	19.37
10% NM2T	723	18.07

Table S5 The leached amounts of Mo and Ni after 24 h of chronopotentiometry measurements for the prepared samples, along with their overpotentials.

Sample	η10 (mV)	Mo leached (µg/L)	Ni leached (µg/L)
NM1	**-**	**103.496**	**4.251**
NM2	**491**	**941.435**	**8.262**
NM3	**-**	**677.094**	**7.366**
1% NM2T	**493**	**353.409**	**6.963**
2% NM2T	**428**	**455.429**	**6.635**
5% NM2T	**395**	**877.32**	**7.877**
10% NM2T	**471**	**196.637**	**3.858**

References

[1] G. Kresse, D. Joubert, From ultrasoft pseudopotentials to the projector augmented-wave method, Phys Rev B 59 (1999) 1758–1775. https://doi.org/10.1103/PhysRevB.59.1758.

[2] G. Kresse, J. Furthmüller, Efficient iterative schemes for *ab initio* total-energy calculations using a plane-wave basis set, Phys Rev B 54 (1996) 11169–11186. https://doi.org/10.1103/PhysRevB.54.11169.

[3] G. Kresse, J. Furthmüller, Efficiency of ab-initio total energy calculations for metals and semiconductors using a plane-wave basis set, Comput Mater Sci 6 (1996) 15–50. https://doi.org/10.1016/0927-0256(96)00008-0.

[4] G. Kresse, J. Hafner, *Ab initio* molecular dynamics for liquid metals, Phys Rev B 47 (1993) 558–561. https://doi.org/10.1103/PhysRevB.47.558.

[5] J.P. Perdew, K. Burke, M. Ernzerhof, Generalized Gradient Approximation Made Simple, Phys Rev Lett 77 (1996) 3865–3868. https://doi.org/10.1103/PhysRevLett.77.3865.

[6] E. Caldeweyher, S. Ehlert, A. Hansen, H. Neugebauer, S. Spicher, C. Bannwarth, S. Grimme, A generally applicable atomic-charge dependent London dispersion correction, J Chem Phys 150 (2019). https://doi.org/10.1063/1.5090222.

[7] P.E. Blöchl, Projector augmented-wave method, Phys Rev B 50 (1994) 17953–17979. https://doi.org/10.1103/PhysRevB.50.17953.

[8] K. Mathew, R. Sundararaman, K. Letchworth-Weaver, T.A. Arias, R.G. Hennig, Implicit solvation model for density-functional study of nanocrystal surfaces and reaction pathways, J Chem Phys 140 (2014). https://doi.org/10.1063/1.4865107.

[9] K. Mathew, V.S.C. Kolluru, S. Mula, S.N. Steinmann, R.G. Hennig, Implicit self-consistent electrolyte model in plane-wave density-functional theory, J Chem Phys 151 (2019). https://doi.org/10.1063/1.5132354.

[10] Kiran Mathew, Rhennig, Javier Bértoli, henniggroup/VASPsol: VASPsol Solvation Module V1.0, (2019).

[11] S. Razzaq, S. Faridi, S. Kenmoe, M. Usama, D. Singh, L. Meng, F. Vines, F. Illas, K.S. Exner, MXenes Spontaneously Form Active and Selective Single-Atom Centers under Anodic Polarization Conditions, J Am Chem Soc 147 (2025) 161–168. https://doi.org/10.1021/jacs.4c08518.

[12] S. Faridi, S. Razzaq, D. Singh, L. Meng, F. Viñes, F. Illas, K.S. Exner, Trends in competing oxygen and chlorine evolution reactions over electrochemically formed single-atom centers of MXenes, J Mater Chem A Mater (2025). https://doi.org/10.1039/D5TA02220G.

[13] K. Dhaka, K.S. Exner, Degree of span control to determine the impact of different mechanisms and limiting steps: Oxygen evolution reaction over Co3O4(001) as a case study, J Catal 443 (2025) 115970. https://doi.org/10.1016/j.jcat.2025.115970.

[14] E. Sargeant, F. Illas, P. Rodríguez, F. Calle-Vallejo, Importance of the gas-phase error correction for O2 when using DFT to model the oxygen reduction and evolution reactions, Journal of Electroanalytical Chemistry 896 (2021) 115178. https://doi.org/10.1016/J.JELECHEM.2021.115178.

[15] K.S. Exner, A Universal Descriptor for the Screening of Electrode Materials for Multiple-Electron Processes: Beyond the Thermodynamic Overpotential, ACS Catal 10 (2020) 12607–12617. https://doi.org/10.1021/acscatal.0c03865.

[16] A.R. Manchuri, K.C. Devarayapalli, B. Kim, Y. Lim, D.S. Lee, Ti3C2 MXene nanosheets integrated cobalt-doped nickel hydroxide heterostructured composite: An efficient electrocatalyst for overall water-splitting, Green Energy & Environment (2024). https://doi.org/10.1016/J.GEE.2024.08.006.

[17] D. Chanda, K. Kannan, J. Gautam, M.M. Meshesha, S.G. Jang, V.A. Dinh, B.L. Yang, Effect of the interfacial electronic coupling of nickel-iron sulfide nanosheets with layer Ti3C2 MXenes as efficient bifunctional electrocatalysts for anion-exchange membrane water electrolysis, Appl Catal B 321 (2023) 122039. https://doi.org/10.1016/J.APCATB.2022.122039.

[18] Y. Sun, Z. Wang, Q. Zhou, X. Li, D. Zhao, B. Ding, S. Wang, Ti3C2 mediates the NiFe-LDH layered electrocatalyst to enhance the OER performance for water splitting, Heliyon 10 (2024) e30966. https://doi.org/10.1016/j.heliyon.2024.e30966.

[19] C. Gan, Y. Zhang, Z. Liu, W. Yan, Q. Jiang, X. Wu, J. Tang, Integrated Co_4MnFe_3/Ti_3C_2: Componential and Structural Engineering toward Boosting Electrocatalytic Oxygen Evolution, The Journal of Physical Chemistry C 125 (2021) 15872–15881. https://doi.org/10.1021/acs.jpcc.1c04892.

[20] K. Zhao, X. Ma, S. Lin, Z. Xu, L. Li, Ambient Growth of Hierarchical FeOOH/MXene as Enhanced Electrocatalyst for Oxygen Evolution Reaction, ChemistrySelect 5 (2020) 1890–1895. https://doi.org/10.1002/slct.201904506.

[21] K. Zhao, X. Ma, S. Lin, Z. Xu, L. Li, Ambient Growth of Hierarchical FeOOH/MXene as Enhanced Electrocatalyst for Oxygen Evolution Reaction, ChemistrySelect 5 (2020) 1890–1895. https://doi.org/10.1002/slct.201904506.

[22] H. Sun, W. Zhang, J.G. Li, Z. Li, X. Ao, K.H. Xue, K.K. Ostrikov, J. Tang, C. Wang, Rh-engineered ultrathin NiFe-LDH nanosheets enable highly-efficient overall water splitting and urea electrolysis, Appl Catal B 284 (2021) 119740. https://doi.org/10.1016/J.APCATB.2020.119740.

[23] J. Jiang, A. Zhang, L. Li, L. Ai, Nickel–cobalt layered double hydroxide nanosheets as high-performance electrocatalyst for oxygen evolution reaction, J Power Sources 278 (2015) 445–451. https://doi.org/10.1016/J.JPOWSOUR.2014.12.085.

[24] Q. Liu, J. Jin, J. Zhang, $NiCo_2S_4$@graphene as a Bifunctional Electrocatalyst for Oxygen Reduction and Evolution Reactions, ACS Appl Mater Interfaces 5 (2013) 5002–5008. https://doi.org/10.1021/am4007897.
